# Core–shell patterning of synthetic hydrogels *via* interfacial bioorthogonal chemistry for spatial control of stem cell behavior†

**DOI:** 10.1039/c8sc00495a

**Published:** 2018-05-24

**Authors:** K. T. Dicker, J. Song, A. C. Moore, H. Zhang, Y. Li, D. L. Burris, X. Jia, J. M. Fox

**Affiliations:** a Department of Materials Science and Engineering, University of Delaware DuPont Hall Newark DE 19716 USA xjia@udel.edu jmfox@udel.edu; b Department of Biomedical Engineering, University of Delaware, Colburn Lab Newark DE 19716 USA; c Department of Mechanical Engineering, University of Delaware, Spencer Lab Newark DE 19716 USA; d Department of Chemistry and Biochemistry, University of Delaware, Brown Lab Newark DE 19716 USA

## Abstract

A new technique is described for the patterning of cell-guidance cues in synthetic extracellular matrices (ECM) for tissue engineering applications. Using *s*-tetrazine modified hyaluronic acid (HA), bis-*trans*-cyclooctene (TCO) crosslinkers and monofunctional TCO conjugates, interfacial bioorthogonal crosslinking was used to covalently functionalize hydrogels as they were synthesized at the liquid–gel interface. Through temporally controlled introduction of TCO conjugates during the crosslinking process, the enzymatic degradability, cell adhesivity, and mechanical properties of the synthetic microenvironment can be tuned with spatial precision. Using human mesenchymal stem cells (hMSCs) and hydrogels with a core–shell structure, we demonstrated the ability of the synthetic ECM with spatially defined guidance cues to modulate cell morphology in a biomimetic fashion. This new method for the spatially resolved introduction of cell-guidance cues for the establishment of functional tissue constructs complements existing methods that require UV-light or specialized equipment.

## Abstract 2

A new technique is described for the patterning of cell-guidance cues in synthetic extracellular matrices.

## Graphical abstract

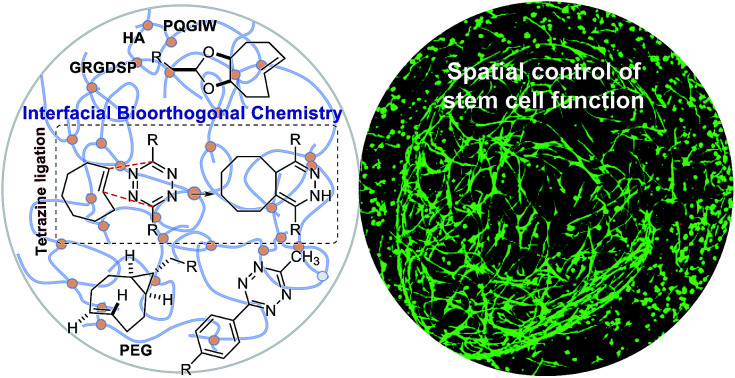

## Introduction

Rational design of synthetic matrices with tissue-specific biochemical compositions and biomechanical properties represents an important step toward the regeneration of functional tissues *in vitro*.^1,2^ Synthetic scaffolds must fulfil the functions of native ECM until cells and produce their own support structures to form an engineered tissue.^3–5^ Hence, it is important to create an adequate cellular microenvironment with spatial variations of mechanical properties, cell-binding motifs, and morphogenic cues in hydrogel scaffolds.^6–8^ Enzyme-mediated chemistry has been used to fabricate multi-layered hydrogels and core–shell particles.^9–12^ Photolithography and stereolithography have enabled spatial tailoring of the cell microenvironment based on photochemistry.^13–18^ For example, a secondary, radical-mediated photo-crosslinking reaction was utilized to spatially tune the stiffness of a hydrogel matrix established by Michael addition.^19^ Visible-light mediated step-growth crosslinking was utilized to produce hydrogels^20^ with multi-layered structures relying on diffusion of the photoinitiator and replenishment of monomer.^21^ Alternatively, photolabile peptide substrates were incorporated in a hydrogel network to afford localized biomolecule tethering through photoactivation.^22^ Finally, the chemical and physical properties of a poly(ethylene glycol) (PEG)-based hydrogel were dynamically and spatially modulated using a nitrobenzyl ether-derived photodegradable functionality.^23^ These materials fabrication strategies generally rely on the use of an external trigger, *e.g.* UV light, to modulate cell morphology and differentiation in a spatial manner. UV-light may not be well tolerated by all cell types and can have depth penetration limitations. Complementary approaches that do not rely on UV-light or specialized equipment, as required for two-photon processes, have the potential to expand the scope of 3D cell culture methods.

Bioorthogonal reactions,^24^ unnatural chemical transformations that occur efficiently in biological context, have recently attracted attention as tools for the fabrication of functional biomaterials.^25,26^ The Diels–Alder reaction has become an important tool for preparing hydrogel matrices for 3D cell culture applications. Furan/maleimide Diels–Alder reactions have been used to create HA based hydrogels that can be subsequently photopatterned with biomolecules using two-photon laser processing.^27–33^ Tetrazine ligation—the inverse-electron-demand Diels–Alder cycloaddition between *s*-tetrazines and strained alkenes, is a rapid bioorthogonal reaction that produces nitrogen gas as the only byproduct.^34–36^ Tetrazine-norbornene ligation has been used to create hydrogels and microparticles with high cyto-compatibility.^37–42^ With *trans*-cyclooctenes (TCO) as the dienophile, this bioorthogonal reaction features fast kinetics, high selectivity at low concentration and compatibility with biological systems.^43,44^ The conformationally strained *trans*-cyclooctenes, *s*-TCO and *d*-TCO, combine with tetrazines with bioorthogonal rate constants surpassed only by sila-*trans*-cycloheptenes.^45,46^ Based on these rapid reactions, we developed diffusion-controlled strategies for the creation of protein-mimetic polymer microfibers^44,47^ and 3D patterned hydrogel spheres,^43^*via* interfacial bioorthogonal polymerization and interfacial bioorthogonal crosslinking, respectively. The fabrication of 3D patterned hydrogels can be carried out in one-step without having to rely on a template or photomask, potentially cytotoxic external triggers,^48^ or step-by-step addition/curing cycles.^49^ Because tetrazine ligation is inherently cytocompatible and specific, it was possible to encapsulate prostate cancer cells during hydrogel formation to produce a cellular construct with high viability. We also demonstrated that our first generation system for diffusion-controlled hydrogel patterning could be used to covalently pattern the hydrogels in 3D with small molecule fluorophores. However, a limitation of our initial system was that only 7% HA modification by tetrazine could be achieved, limiting the method to the creation of only soft hydrogels, and prohibiting the ability to spatially control modulus or presentation of cell guidance cues in the matrix.

**Fig. 1:**
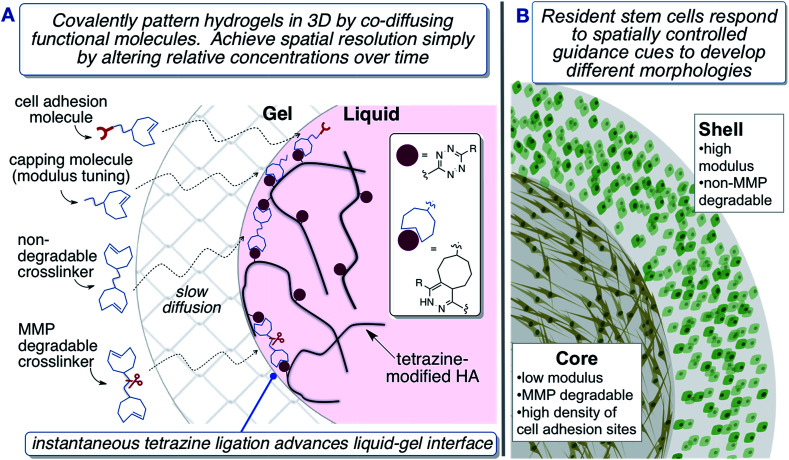
Fabrication of biomimetic hydrogels with a 3D core–shell pattern to provide spatial guidance cues to encapsulated hMSCs. (A) TCO-conjugated molecules diffuse across the crosslinked shell to react at the gel–liquid interface. (B) hMSCs adopt different morphologies depending their spatial localization within the matrix.

**Fig. 2:**
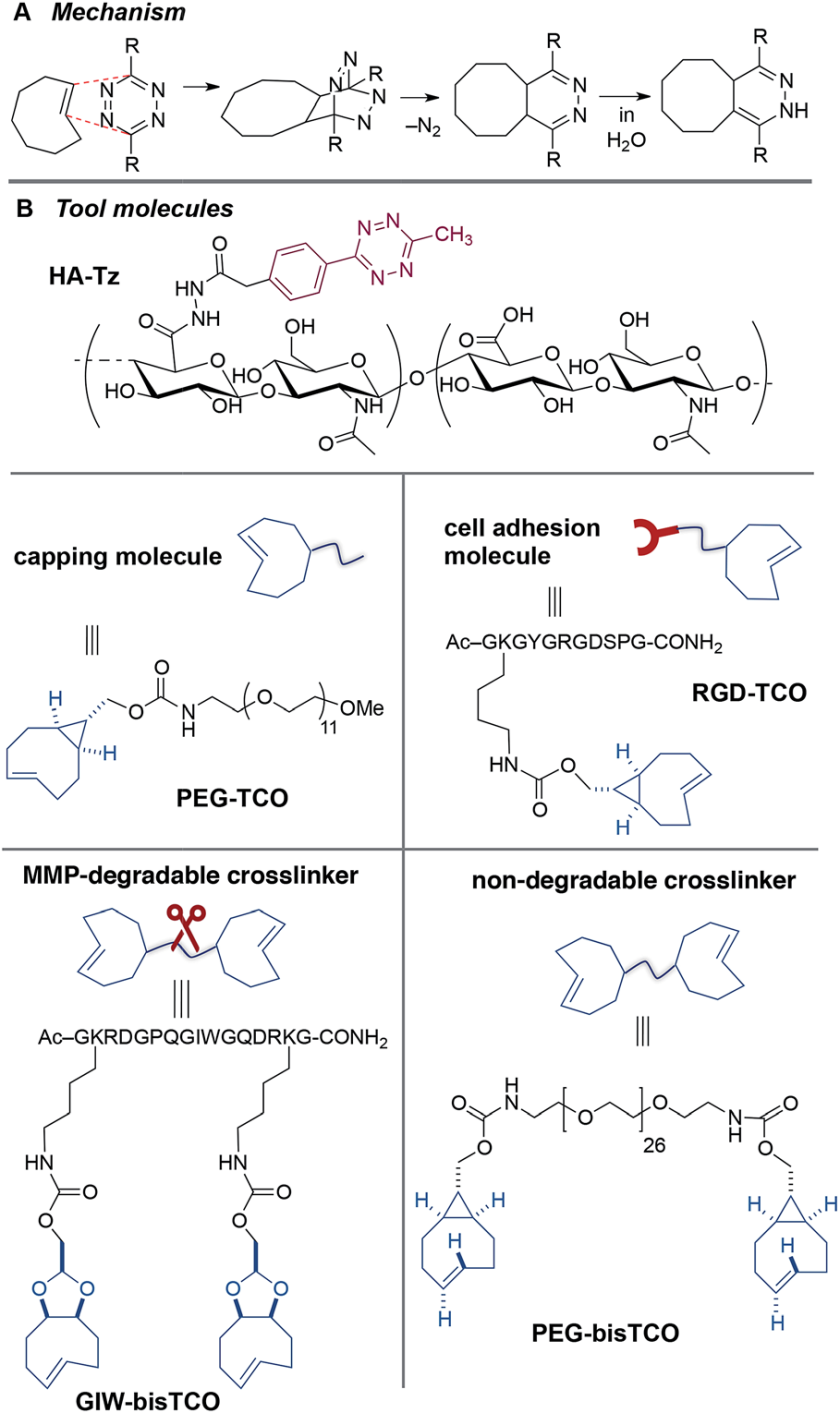
Synthetic toolbox for core–shell patterning. (A) Tetrazine-TCO ligation mechanism. (B) Hydrogel building blocks include tetrazine-modified HA (HA-Tz), non-degradable and MMP-degradable TCO crosslinkers (PEG-bisTCO and GIW-bisTCO) and monofunctional molecules (PEG-TCO, and RGD-TCO).

Described herein is a second generation system for interfacial bioorthogonal crosslinking which enables the engineering of biomimetic hydrogels with a 3D core–shell structure that can spatially dictate the behaviour of encapsulated hMSCs (Fig. 1). Hydrogels were fabricated using tetrazine-modified hyaluronic acid (HA-Tz) along with mono- and bi-functional TCO-derivatives (Fig. 2) *via* a diffusion-controlled interfacial crosslinking mechanism. Key to the design was use of a 3-methyl-6-aryl-*s*-tetrazinyl hydrazide, which is more nucleophilic and enabled much higher levels of HA functionalization, and consequently enabled the ability to spatially tune the modulus and presentation of ligands in resultant gels. Time-dependent alteration of the TCO reservoir composition resulted in the creation of hydrogels with a 3D core–shell structure. Biochemical signals, including matrix metalloprotease (MMP)-degradable peptide and integrin binding motifs, and biomechanical cues were patterned into the hydrogels to spatially direct cellular behavior.^50,51^ We envision that this synthetic platform should ultimately be useful for the engineering of complex tissues with layered structures.^1^

## Results and discussion

### Hydrogel synthesis and fluorescent tagging

Hydrogel precursors were derived from defined-length PEG, bioactive peptides, and HA, a natural non-sulfated glycosaminoglycan abundant in connective tissue ECM.^52–54^ Thus, high molecular weight HA (430 kDa) was coupled with Tz-hydrazide through 1-ethyl-(3,3-dimethylaminopropyl) carbodiimide (EDC)-mediated coupling chemistry at pH 4.75 to yield a tetrazine-functionalized HA (HA-Tz, Fig. 2B) with 18 mol% tetrazine incorporation, as quantified by UV-vis spectroscopy (Fig. S1†) and ^1^H NMR and (Fig. S11†). Separately, *s*-TCO nitrophenyl carbonate was combined with PEG_27_-diamine to produce a non-degradable crosslinker (PEG-bisTCO, Fig. 2B) with a molecular weight of 1.6 kDa. Degradability of the matrix by cell-secreted proteases is desirable to maintain cell viability and to enhance cell spreading and migration.^55,56^ Accordingly, an MMP-cleavable peptide with a basic sequence of GPQG↓IWGQ flanked with charged amino acid residues (abbreviated as GIW) was modified with *d*-TCO nitrophenyl carbonate through lysine amines to produce GIW-bisTCO (2.2 kDa, Fig. 2B). To enable spatial control of gel mechanics in a diffusion-controlled manner, a mono-functional ‘capper’ molecule was synthesized by conjugating *s*-TCO to the amino end of *m*PEG_12_-NH_2_ (PEG-TCO, Fig. 2B). To introduce cell adhesive ligands to the synthetic matrix, *s*-TCO was conjugated to the GKGYGRGDSPG peptide through the lysine amine (RGD-TCO, 1.3 kDa, Fig. 2B). Our previous investigation^21^ showed that such derivation does not compromise the cells' ability to bind to the RGD peptide.

Covalently crosslinked hydrogels were fabricated *via* the addition of an HA-Tz droplet into a reservoir containing mono- and bifunctional TCO molecules. The bioorthogonal nature of tetrazine ligation (Fig. 2A) allows for the hydrogel to be formed under physiological conditions in either phosphate buffered saline (PBS) or cell culture media. Stopped-flow experiments (Fig. S2†) conducted in water at 25 °C revealed that PEG-TCO and PEG-*d*-TCO reacted with Tz-hydrazide with a second order rate constant, *k*_2_, of 6.70 × 10^4^ M^−1^ s^−1^ and 9.94 × 10^3^ M^−1^ s^−1^, respectively. Thus, both *s*-TCO and *d*-TCO react with HA-Tz with rates that are more rapid than the rate of diffusion through a crosslinked hydrogel. As depicted in Fig. 1A, as soon as a droplet of HA-Tz (5% in PBS) was added to the TCO reservoir, a crosslinked shell formed instantaneously between the two liquids. Over the course of 4 h, the low molecular weight TCO species continued to diffuse across the crosslinked shell to react with the high molecular weight HA-Tz at the gel–liquid interface, crosslinking the droplet radially towards the core, until all tetrazine sites on HA were consumed. The resultant hydrogels had a diameter of 2.5–2.9 mm (Table 1).

**Table 1:** Preparation of HA-based hydrogels with varying stiffness, degradability and adhesivity

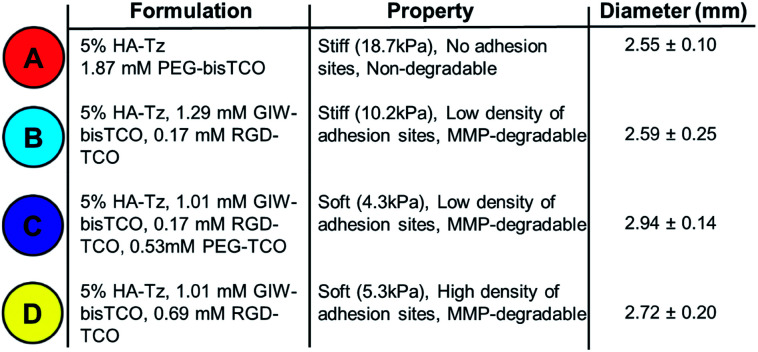

**Fig. 3:**
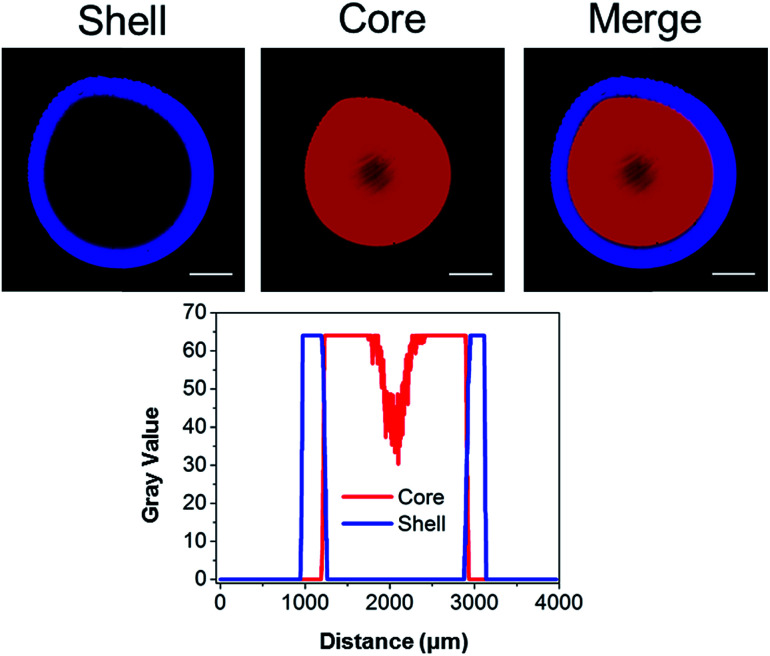
Covalent tagging of fluorescent dyes in a spatial core–shell pattern. (Top) confocal microscopy images of the hydrogel showing a distinct core–shell structure (central slice). (Bottom) intensity plot across the gel showing the presence of a sharp interface between the core and the shell regions. Scale bar: 500 μm.

We previously illustrated that diffusion-controlled kinetics can be used to pattern hydrogels in 3D.^43^ To validate our second generation system, fluorescent TCO-conjugates of Cy3 (Ex: 555 nm; Em: 565 nm) and Alexa Fluor® 647 (Ex: 650 nm; Em: 665 nm) were used to covalently tag the gels during the crosslinking process. At time zero, a droplet of HA-Tz was introduced to a crosslinking reservoir containing Alexa-TCO^18^ (3 μM) and PEG-bisTCO (1.87 mM). The crosslinking reaction was allowed to proceed for 1 h, and the reservoir was switched to one containing Cy3-TCO (6 μM) and PEG-bisTCO (1.87 mM). The mixture was left undisturbed at ambient temperature for additional 3 h to produce a fully crosslinked hydrogel. As shown in Fig. 3, Alexa was covalently tagged only to the outer shell of the hydrogel (0.3 mm) during the initial 1 h crosslinking, whereas Cy3 was incorporated only into the hydrogel core during the remaining 3 h. There was also a sharp interface where the Alexa signal stopped and the Cy3 signal started, confirming that the fluorescent tags are introduced simultaneously with crosslinking at the gel–liquid interface. Thus, changing the TCO reservoir composition as a function of time provides a simple way to covalently pattern hydrogels in 3D. As shown below, the second generation system for hydrogel patterning also permits 3D tuning of gel mechanics, cell adhesivity and matrix degradability.

### Bioorthogonal tuning of hydrogel properties

**Fig. 4:**
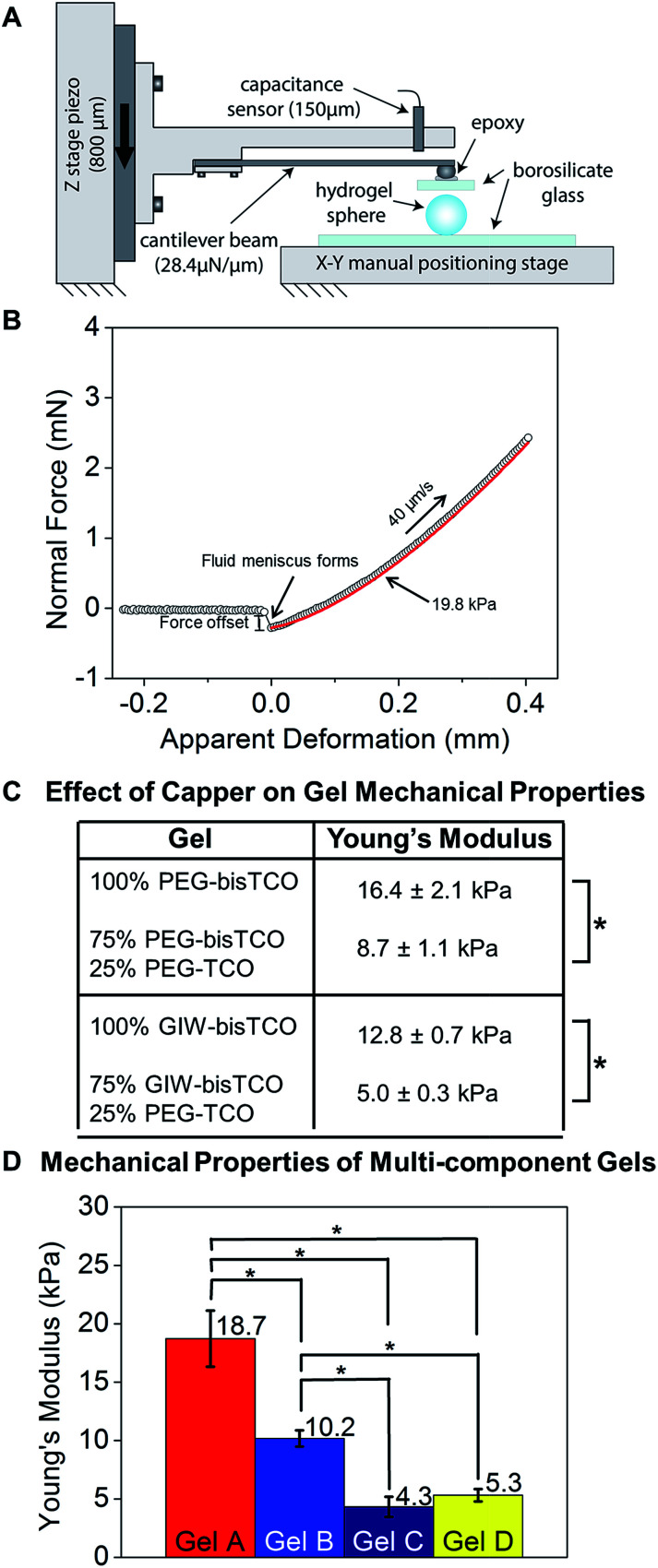
Characterization of hydrogel mechanical properties using a custom-built, micro-materials tester. (A) Schematic of the micro-materials tester used in this study. The hydrogel rested on the lower glass flat was compressed by a borosilicate plate. (B) Representative force–displacement curve demonstrating the top plate approach, contact with and compress the hydrogel. (C) Young's modulus of hydrogels prepared using different and mono- and bis-TCO derivatives. Hertzian model of parallel plate compression was used and Young's modulus was calculated assuming a Poisson ratio of 0.5. The crosslinking reservoir contained 0% or 25% monofunctional TCO capper. (D) Young's modulus of hydrogels formulated according to Table 1 and used for subsequent cell culture studies. *p < 0.05.

Mechanical properties of hydrogels can directly influence cellular behaviors.^57–59^ Under 2D culture, hMSCs cultured on softer hydrogels have been shown to differentiate into a neural phenotype while those cultured on stiffer substrates tended to go towards an osteoblastic fate.^60,61^ Previously, hydrogel stiffness has been altered by varying the concentration or the functionality of the macromers or crosslinkers.^62,63^ In our system, matrix stiffness was tuned by changing the ratio of mono-functional TCO capper and bifunctional TCO crosslinker, both exhibiting a similar diffusivity. The capper molecule consumes tetrazine groups on HA to generate network defects as a dangling chain.^64^ As such, it effectively removes reactive sites that would otherwise contribute to the establishment of elastically active connections. The mechanical properties of the hydrogels were quantified by compression experiments using a custom micro-materials tester (Fig. 4A, Video S1†) under hydrated conditions.^65^ Normal force was measured as a function of compression and the resulting compression response (based on the approaching curve only) was fit to a Hertzian model of parallel plate compression of an elastic sphere (Fig. 4B). The compressive modulus was converted to Young's modulus, assuming a Poisson ratio of 0.5.^66^

As shown in Fig. 4C and S6,† we demonstrated that inclusion of a PEG-TCO capping molecule in the bisTCO reservoir resulted in the reduction of stiffness for gels crosslinked by PEG-bisTCO or GIW-bisTCO. Hydrogel samples prepared using 100% PEG-bisTCO (1.87 mM) or GIW-bisTCO (1.35 mM) had a Young's modulus of 16.4 ± 2.1 kPa and 12.8 ± 0.7 kPa, respectively (Fig. 4C, S5 and S6†). The equilibrium swelling ratio was found to be 25 ± 4 and 39 ± 5 for gels crosslinked with PEG-bisTCO and GIW-bisTCO, respectively. The MMP-degradable network swells more due to the large number of charged amino acid residues in the peptide sequence. Inclusion of monofunctional PEG-TCO in the bisTCO reservoir produced hydrogels that were significantly softer. At a 1/4 capper (PEG-TCO)/crosslinker (PEG-bisTCO or GIW-bisTCO) ratio, an average Young's modulus of 8.7 ± 1.1 kPa and 5.0 ± 0.3 kPa were detected for non-degradable and MMP-degradable networks, respectively (Fig. 4C and S5 and S6†).

Having shown that capping molecules could be used to tune the moduli of gels produced through interfacial crosslinking, we next sought to demonstrate that interfacial chemistry could be used to create more complex gels with varying stiffness, MMP-degradability and cell adhesivity. Four gel types, designated as Gels A, B, C, and D, were produced employing gel formulations outlined in Table 1 and the mechanical properties (Fig. 4D) were analysed as described above. Gel A was found to be the stiffest with a Young's modulus of 18.7 ± 2.4 kPa. Gel B made with 5% RGD-TCO and 95% GIW-bisTCO was softer, and had an average Young's modulus of 10.2 ± 0.7 kPa. Gels C and D had 25% mono-functional TCO molecules, thus were the softest with moduli of 4.3 ± 0.9 kPa and 5.3 ± 0.5 kPa, respectively. Having a similar molecular weight, PEG-TCO and RGD-TCO had a comparable capacity in modulating gel stiffness. Statistically, gel A was significantly stiffer than gels B–D (*p* < 0.05), whereas gel C and D had comparable stiffness (*p* > 0.05). These results further corroborate the ability to tune gel stiffness by introducing the mono-functional capper molecule.

The ability of cells to breakdown their ECM is an important prerequisite for maintaining proper cell functions.^62,67^ In covalently crosslinked 3D hydrogels, stem cell fate is directly related to the ability of cells to generate traction forces, through MMP-mediated matrix degradation, independent of cell morphology and matrix stiffness.^68^ Hydrogel degradation was monitored gravimetrically with or without type IV collagenase. When incubated in Hank's balanced salt solution (HBSS) containing 100 U mL^−1^ collagenase at pH 7.4, a significant mass loss was observed within 30 min for gels crosslinked with GIW-bisTCO, and the gels were completely disintegrated within 1 h (Fig. S8†). Initially, hydrogels were buoyant and maintained the spherical shape. By 30 min, hydrogels had lost the spherical shape and conformed to the bottom of the glass cylinder as a dome. By 60 min, hydrogels were fully degraded. By contrast, gels incubated in enzyme-free media were intact. No mass loss was detected from gels made with PEG-bisTCO and incubated with or without the enzyme. These results confirmed the specificity of enzymatic degradation and the absence of hydrolytic degradation within the period of the experiment.

### 3D cell culture

**Fig. 5:**
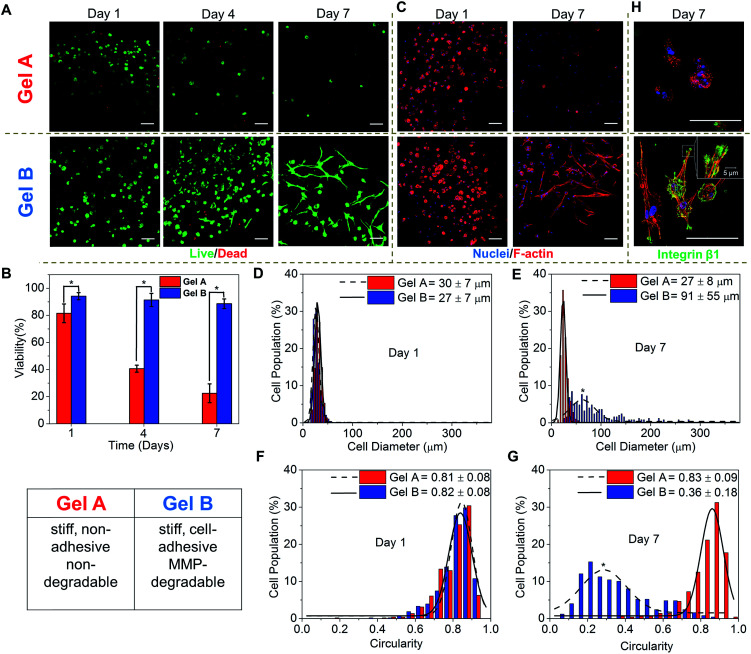
3D culture of hMSCs in homogeneous hydrogels prepared using either PEG-bisTCO (Gel A) or GIW- and RGD-TCO (Gel B). (A) Confocal images of 3D cultures stained by calcein AM (green) and ethidium homodimer (red) for live and dead cells, respectively, after 1, 4 and 7 days of culture. (B) Quantification of cell viability based on live/dead assay using ImageJ. (C) Confocal images of 3D cultures stained by F-actin (red) and DAPI (blue) after 1 and 7 days of culture. (D–G) Characterization of cell morphology by Feret diameter (D and E) and circularity (F and G) using ImageJ. (H) Higher magnification (40×) confocal images of hMSCs stained for DAPI (blue), F-actin (Red) and integrin β1 (green) after 7 days of culture. *p < 0.05. Scale bar: 100 μm.

For 3D cell encapsulation, hMSCs were first dispersed in an HA-Tz (5% in PBS) solution and the cell suspension was dropped into a TCO reservoir containing PEG-bisTCO (1.87 mM) only or RGD-TCO (0.17 mM) and GIW-bisTCO (1.29 mM) to establish cell-laden constructs of Gel A and Gel B, respectively. The reaction was complete in 4 h at 37 °C, as determined by the disappearance of the tetrazine chromophere,^43^ before the TCO reservoir was replaced with fresh hMSC growth media. The 3D cultures were maintained for up to 7 days and cell viability was assessed by counting live (green) and dead (red) cells from fluorescently stained constructs under confocal microscope (Fig. 5A and B). After 1 day of culture, hMSCs encapsulated in PEG-crosslinked hydrogels had a viability of 80%, confirming the cytocompatible nature of the hydrogel and crosslinking chemistry. Replacing the PEG crosslinker with an MMP-degradable substrate, at the same time introducing RGD, significantly improved the overall viability (94%), highlighting the importance of including key cell-responsive motifs in synthetic matrices. Prolonged culture of hMSCs in Gel A, a non-degradable covalent network, led to a progressive decrease in cell viability, in agreement with previous observations.^69^ On the other hand, cells encapsulated in the cell-adhesive and MMP-degradable hydrogels maintained high viability (>90%) throughout the 7 day culture period. The ability of hMSCs to breakdown their matrix through MMP secretion^56,67^ led to a significant change in cell morphology, from a rounded shape at day 1 to a spindle shape with long cellular processes by day 7 (Fig. 5A and B).

It is well documented that cell shape is a potent regulator of cell survival, growth, signalling, differentiation and tissue development.^70,71^ Therefore, establishing biomimetic microenvironment that regulates cell morphology is a powerful strategy for controlling cell physiology, a first step towards the establishment of engineered tissues or tissue models. Thus, the morphology of hMSCs residing in blank (Gel A) or bioactive (Gel B) hydrogels were analysed by immunofluorescent staining and confocal imaging. After 1 day of culture, cells in both types of hydrogels exhibited a similar, rounded cell shape, with a diameter, and circularity of 27–30 μm, and 81–82%, respectively (Fig. 5C–G). By day 7, cells in the two types of gels had developed significantly different cell morphology. Cells in the non-adhesive and non-degradable gels (Gel A) mostly maintained the rounded shape, although a slight decrease diameter was observed, potentially as a result of cells undergoing apoptosis (Fig. 5C–G). By day 7, only 22 ± 7% cells were viable. hMSCs were distributed in the blank gels as single, rounded cells with distinct cortical actin, with little expression of integrin β1 (Fig. 5H), a protein of the β subunit which plays a prominent role in RGD attachment and cell motility.^72^ By contrast, cells in MMP-degradable/cell-adhesive gels became more elongated and spread-out, having an average diameter and circularity of 91 ± 55 μm and 36 ± 18%, respectively (Fig. 5C–G). Cells developed polarized, spindle-shaped morphology with bundles of F-actin stress fibres distributed throughout the entire cellular extensions (Fig. 5C and H). Integrin clustered at the edges of the elongated actin stress fibres, as punctate foci, where they attach to the hydrogel matrix (Fig. 5H). Organization of actin monomer into stress fibres indicates active actin polymerization events leading to the development of load-bearing cell–matrix binding that is conducive to cell extension in 3D. hMSCs were also encapsulated in a synthetic matrix that exhibited a similar stiffness and RGD density to Gel B, but was not susceptible to MMP-mediated degradation. After 7 days of culture, no significant change in cell morphology was observed. Cells remain rounded during the 7 day culture, with no obvious F-actin polymerization (Fig. S9†). Our observation is in agreement with earlier reports that, when entrapped in a covalent network with cell-adhesive ligands, cell-mediated matrix degradation is essential to promote integrin-mediated^73^ cell–matrix interactions. Matrix mechanics might also play a role here as the blank, non-degradable Gel A is significantly stiffer than the bioactive, degradable Gel B (Fig. 4D).^74–76^

As a preliminary assessment of cell function, we conducted additional experiments to analyse cellular secretion of type I collagen, an important structural protein found in the ECM. Our results (Fig. S10†) show that collagen expression is weak and diffuse by the rounded cells cultured in blank Gel A. Intense collagen staining was observed from cells cultured in bioactive Gel B. Collagen deposition along the intracellular stress fibers is obvious. In agreement with our previous observations,^77^ immunostaining of collagen I expressed by MSCs appears cytoplasmic, *i.e.* within the secretory pathway.

### Spatial control of stem cell behaviour

**Fig. 6:**
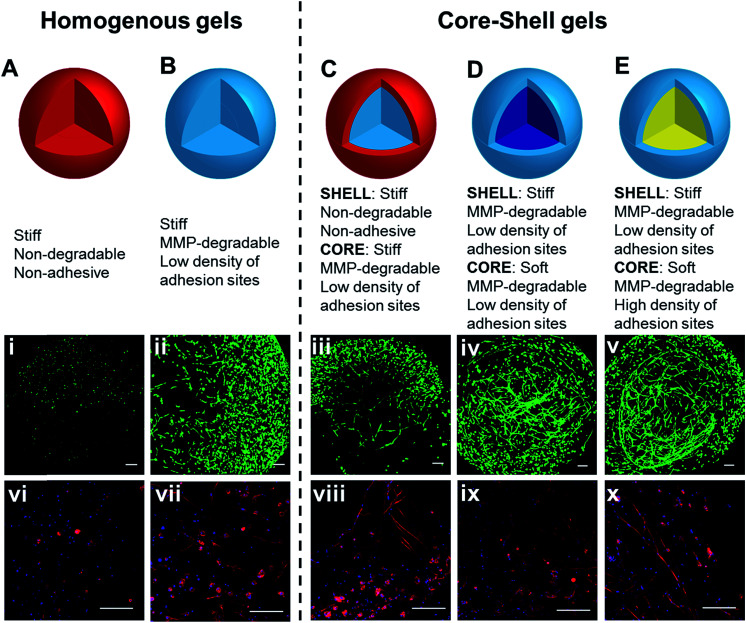
3D culture of hMSCs in hydrogels with bioactive core–shell patterning. Cells grown in homogeneous gels (A and B), prepared using (A) PEG-bisTCO or (B) GIW-bisTCO and RGD-TCO (0.17 mM), were included for comparison purposes. Hydrogels with a core–shell pattern (C–E) were created following conditions outlined in Table 1. Construct in (C), prepared using PEG-bisTCO and GIW-bisTCO/RGD-TCO had a blank shell (red) and a bioactive core (light blue). Construct in (D), prepared using GIW-bisTCO/RGD-TCO with or without the PEG-TCO capper had a stiff shell (light blue) and a softer core (dark blue). Construct in (E), prepared using GIW-bisTCO with low and high concentrations of RGD-TCO, had a stiffer, less adhesive shell and a softer, more adhesive core. Cultures were maintained for 7 days before staining and confocal imaging. (i–v) Live and dead cells were stained green and red, respectively. (vi–x) F-actin and nuclei were stained red and blue, respectively. Scale bar: 200 μm.

Having confirmed the distinctly different cellular responses to blank (Gel A) and bioactive (Gel B) gels, we next spatially patterned the two matrix compositions into an individual hydrogel *via* diffusion controlled interfacial crosslinking. One hour after HA-Tz was added to the PEG-bisTCO reservoir, the partially crosslinked gel was transferred to a reservoir containing GIW-bisTCO (1.29 mM) and RGD-TCO (0.17 mM) and the reaction was allowed to proceed for 3 h to establish a fully crosslinked hydrogel. As inferred from the dye labelling experiment (Fig. 3), the resultant matrix exhibited a core–shell structure, having a core of approximately 1.7 mm in diameter encased by a shell of 0.3 mm thick. Based on mechanical evaluations of the homogeneous gels (Fig. 4D), the outer shell was 1.85 ± 0.25 times stiffer than the inner core. Again, hMSCs cultured in the homogenous and bioinert gel (Gel A) remained round after 7 days (Fig. 6A) while those cultured in the homogenous and bioactive hydrogel (Gel B) exhibited a spread-out morphology with elongated stress fibres (Fig. 6B). Cells elongated along a single axis in 3D to establish an interconnected cellular mesh by day 7. When these two gel compositions were patterned into the same hydrogel in a core–shell geometry, cells maintained the respective shapes in the corresponding regions (Fig. 6C). Intriguingly, cells at the core–shell boundary were interconnected and those in the outer shell crosslinked with PEG-bisTCO developed inward projections towards this boundary. Because cells residing in the outer shell were unable to degrade and attach to their matrix, they could not migrate to the more favourable region. Instead, they aligned their cell bodies toward the more permissive centre, likely as a result of communication with cells in the core through paracrine signaling.^78^ Although cells could only remodel the GIW-containing core, hyaluronidase secreted by cells residing in the outer layer may contribute to the development of long projects at the boundary.

Biomechanical cues can be similarly presented in a core–shell fashion in the hydrogels using PEG-TCO capper, along with GIW-bisTCO crosslinker. One hour after HA-Tz was added to the TCO reservoir containing GIW-bisTCO and RGD-TCO, the partially crosslinked hydrogel was transferred to a reservoir containing PEG-TCO and bisTCO at a molar ratio of 1 : 4 to complete the crosslinking. RGD-TCO concentration was maintained constant (0.17 mM) throughout the entire crosslinking process. Thus, the resultant gel had a core and shell with properties of Gel C and B, respectively. As inferred from Fig. 4D, the shell was 2.45 ± 0.57 times stiffer than the central core. Because all crosslinks were MMP-degradable, cells in both core and shell regions were able to spread (Fig. 6D). The ability of cells to perceive the difference in their environment was reflected again by the different morphologies cells adopt. The loosely crosslinked core promote a more rapid spreading and foster the development of longer cellular extensions. By day 7, cells in the more densely crosslinked shell region had just started to extend, and their cell bodies were relatively small.

While decreased stiffness can be achieved through introduction of the PEG-based capper molecule without altering other properties, the same effect can be achieved using RGD-TCO, which essentially functions as a capper from the mechanics perspective. One hour after HA-Tz was added to a reservoir containing GIW-bisTCO (95%) and RGD-TCO (5%), the partially crosslinked hydrogel was transferred to a reservoir with an increased concentration of RGD-TCO (25%) along with GIW-bisTCO (75%) to finish crosslinking. Consequently, the core with a composition of Gel D was softer (1.93 ± 0.23 times) and more cell-adhesive than the shell of Gel B. As shown in Fig. 6E, hMSCs formed a similar core–shell like structure, with a network of highly spread-out cells residing at the boundary between the core and shell after 7 days of culture. The softer core had a lower GIW concentration, but a higher RGD concentration. In matrices with higher concentrations of RGD peptide, hMSCs show high viability and proliferation as the adhesion supports cells during ECM degradation. Unlike the previous case, cells at the core–shell boundary were aligned along the circumferential direction, rather than projecting inwards. We speculate that, in this case, because cells in both core and shell can spread, there was no directionality in cytokine secretion.

As shown here, interfacial tetrazine ligation provides a powerful, new method for modulating the biochemical and biomechanical properties of synthetic ECMs. Without any specialized equipment, 3D spatial patterning was achieved *via* the timed alteration of the TCO bath composition. Our work was motivated by the need to create ECM templates for the engineering of mechanically active soft tissues that exhibit characteristic gradient or layered structures, as a consequence of the mechanical roles these tissues perform. Under the influence of the combined biochemical and mechanical factors, cells residing in these tissues exhibit different behaviours depending on their spatial localization within the tissue. Here, we demonstrated the development of a cell-instructive synthetic ECM displaying layered structures to provide the resident stem cells with spatially controlled guidance cues. With further development, we expect that the resident stem cells will actively remodel the synthetic environment and deposit natural matrix components in a spatial fashion reflecting that of the original ECM template. We anticipate that the new method for establishing spatial control of stem cell behaviour presented here should find applications in the tissue repair, remodelling and regeneration.

## Conclusions

We have demonstrated the use of interfacial bioorthogonal chemistry for the preparation of spatially patterned hydrogels with distinct chemical and mechanical microenvironments. Through temporally controlled introduction of *trans*-cyclooctene (TCO) conjugates during the crosslinking process, the enzymatic degradability, cell adhesivity, and mechanical properties of the synthetic microenvironment can be tuned with spatial precision. hMSCs encapsulated in the bioactive region were able to degrade their matrix to adopt a spread morphology while those in the blank, non-degradable region remained round. The bioorthogonal platform allows straightforward patterning of cellular microenvironments to trigger desired responses or to promote the formation of multilayer tissues.

## Experimental

### General considerations

All reactions were carried out in glassware that was flame-dried under vacuum and cooled under nitrogen. Cy3-TCO was purchased from AAT Bioquest. *O*,*O*′-Bis(2-aminoethyl)hexacosaethylene glycol (≥95% oligomer purity) was purchased from Santa Cruz Biotechnology. Hyaluronic acid (sodium salt, 430 kDa) was a generous gift from Sanofi/Genzyme Corporation. Reactive intermediates or products, including (rel-1*R*,8*S*,9*R*,4*E*)-bicyclo[6.1.0]non-4-ene-9-ylmethanol,^45^*d*-TCO-carbonate,^79^ RGD-TCO^47^ and Alexa-TCO^43^ were prepared following known procedures. Peptides were synthesized using CEM Liberty Blue Peptide Synthesizer. Dialysis membranes were purchased from Spectrum Labs (MWCO: 10 kDa). Flash chromatography was performed using normal phase Silicycle silica gel (40–63 d, 60 Å). Other reagents were purchased from commercial sources without additional purification. The detailed synthesis of hydrogel precursors can be found in the ESI.†

### Hydrogel synthesis

HA-Tz and the bisTCO crosslinker (PEG-bisTCO or GIW-bisTCO) were separately dissolved in PBS at a concentration of 5 wt% and 0.3 wt%, respectively. Next, HA-Tz was dropped *via* a 25G syringe into the bis-TCO solution (300 μL for PEG-bisTCO and 414 μL for GIW-bisTCO) in a 48 well plate (BD Falcon™). The interfacial crosslinking process was allowed to occur at 37 °C for 4 h without any agitation until the tetrazine chromophore (pink) disappeared from the droplet. The bis-TCO solution was then replaced with fresh PBS. Hydrogels with a lower crosslinking density were prepared *via* the incorporation of PEG-TCO in the crosslinking bath at a bisTCO-to-monoTCO molar ratio of 4/1. Homogeneous matrices (Gels A–D) used for 3D culture studies were prepared similarly by adding HA-Tz to a TCO reservoir with a composition depicted in Table 1. Hydrogels with a core–shell structure were prepared by incubating the HA-Tz droplet in the first reservoir for 1 h to establish the shell, followed by a 3 h exposure to a second reservoir to complete the core.

### Hydrogel swelling and degradation

The as-synthesized hydrogels were dehydrated in graded ethanol solutions and vacuum dried. The swelling ratio, reported as an average of three repeats, was determined as the ratio of the initial weight of the wet gel to the weight of the dry product. For degradation studies, hydrogels were washed with HBSS and allowed to equilibrate overnight. Hydrogels were then weighed to record the starting mass. Hydrogels were then placed in HBSS solutions with or without 100 U mL^−1^ Collagenase Type IV. The supernatant was removed and the gel mass was recorded every 30 min for up to 4 h. Values were normalized to the starting mass.

### Fluorescent tagging

HA-Tz (5 wt% PBS) was dropped into a reservoir containing PEG-bisTCO (1.87 mM) and Alexa-TCO (3 μM). The reaction was allowed to proceed at 37 °C for 1 h. The partially crosslinked gel was transferred to a different reservoir containing PEG-bisTCO (1.87 mM) and Cy3-TCO (6 μM). The reaction was allowed to continue for an additional 3 h. The hydrogels were washed with PBS overnight before imaging using a Zeiss 710 NLO confocal microscope with a 5× objective.

### Mechanical properties

Compression experiments were conducted using a custom-made micro-materials tester,^34^ modified for parallel plate ramp compression of hydrogels (Fig. 4A). The parallel plates were made of 1 mm thick borosilicate glass microscope slides (12-550-A3, Fisher Scientific). One glass flat was fixed to an XY table while the other was fixed to the free end of a calibrated cantilever beam load cell with a μN resolution. The fixed end of the beam was driven by a piezoelectric stage (0–800 ± 0.002 μm). Hydrogels were placed on the lower glass flat and a small droplet of PBS was placed at the base. Prior to contact, the hydrogel was aligned using the XY table and positioned 200 μm below the moving glass flat. Subsequently, the piezoelectric stage was driven toward the sample over a travel range of 700 μm at a rate of 45 μm s^−1^; the compression rate of the sample was lower at 40 μm s^−1^ due to deformation of the load cell. Compressive modulus was determined based on the approaching curve using the Hertzian model, and was converted to Young's modulus based on the assumed Poisson ratio of 0.5.

### Cell maintenance and 3D culture

hMSC cells were maintained in a MSC growth BulletKit medium (Lonza, Walkersville, MD). HA-Tz was dissolved in the PBS at a concentration of 5 wt% and was sterilized by exposure to germicidal UV light for 15 min. All TCO-conjugated molecules were dissolved in PBS and sterile-filtered using a 0.22 μm poly(vinylidene fluoride) (PVDF) syringe filter (Thermo Fisher Scientific, Waltham, MA). Cellular constructs were prepared following procedures described above for hydrogel synthesis and using HA-Tz containing suspended hMSCs (1 × 10^6^ mL^−1^). Upon completion of the crosslinking reaction, the constructs were transferred to wells containing fresh media and were incubated at 37 °C for up to 7 days, with media refreshed every other day.

### Cell viability

Percent cell viability was assessed by live/dead staining using calcein AM and ethidium homodimer after 1, 4 and 7 days of culture. Short z-stacks of 105 μm with 15 μm slices were taken with Zeiss 710 NLO confocal microscope with a 10× objective. The images were flattened in Zeiss's Zen software to produce maximum intensity projections. By using Image J and counting the live and dead cells in each image, percent viability was quantified. Values are presented as a percentage of live cells compared with the total number of cells.

### Cell morphology

Cell morphology was assessed by staining hydrogel constructs after 1 and 7 days of culture for F-actin using Alexa Fluor 568 phalloidin, with the nuclei counter stained by DAPI, following our previous protocols.^80^ Selected samples were incubated with primary integrin β1 antibody (Santa Cruz Biotechnology) at a 1 : 100 dilution in 1× PBS containing 3% BSA for 2 h at room temperature. Samples were then treated with Alexa Fluor 488-conjugated secondary antibody at a 1 : 200 dilution in the same buffer for 2 h at room temperature. Stained samples were imaged using a Zeiss 710 NLO confocal microscope with a 10× objective. Short z-stacks of 106.7 μm with 6.2 μm slices were taken and converted into maximum intensity projections using Zen. Using Image J, cell body was accessed for total area, Feret's diameter, circularity and roundness. Values were plotted as a histogram with a fitted Gaussian curve.

### Statistical analysis

All quantitative analyses were performed in triplicate and results were expressed as the mean ± standard deviation. Statistical significance was evaluated by analysis of variance (two-way ANOVA), followed by Tukey–Kramer post-hoc test. A *p*-value of <0.05 was considered to be statistically different.

## Conflicts of interest

There are no conflicts to declare.

## Supplementary Material

SC-009-C8SC00495A-s001

SC-009-C8SC00495A-s002
