# Supplementary material for: Core–shell patterning of synthetic hydrogels via interfacial bioorthogonal chemistry for spatial control of stem cell behavior
Source: Chem Sci. 2018 May 24;9(24):5394–404. doi: 10.1039/c8sc00495a (PMC6009435; doi:10.1039/c8sc00495a)
Supplement: SC-009-C8SC00495A-s001 [file SC-009-C8SC00495A-s001.pdf]

Electronic Supplementary Material (ESI) for Chemical Science.
This journal is © The Royal Society of Chemistry 2018

Supplementary Information

Core-Shell Patterning of Synthetic Hydrogels via Interfacial Bioorthogonal Chemistry for Spatial Control of Stem Cell Behavior

K. T. Dicker,[a,‡] J. Song,[a,‡] A. C. Moore,[b] H. Zhang,[d] Y. Li,[d] D. L. Burris,[b,c] X. Jia*[,a, b] and J. M. Fox*[,a,d]

[a]Department of Materials Science and Engineering, University of Delaware, DuPont Hall, Newark, DE 19716

[b]Department of Biomedical Engineering, University of Delaware, Colburn Lab, Newark, DE 19716

[c]Department of Mechanical Engineering, University of Delaware, Spencer Lab, Newark, DE 19716

[d]Department of Chemistry and Biochemistry, University of Delaware, Brown Lab, Newark, DE 19716

1. Synthetic Procedures.

1.1. Synthesis of 2-(4-(6-methyl-1,2,4,5-tetrazin-3-yl)phenyl)acetohydrazide (Tz-hydrazide)

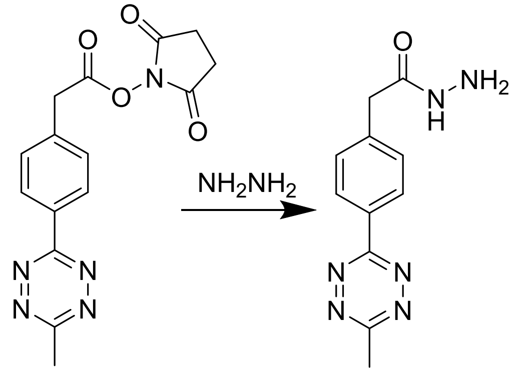

Hydrazine anhydrous (73.4 mg, 2.29 mmol) dissolved in dichloromethane (3 mL) was slowly added a solution of 2,5-dioxopyrrolidin-1-yl 2-(4-(6-methyl-1,2,4,5-tetrazin-3-yl)phenyl)acetate (purchased from Kerafast, 150 mg, 0.46 mmol) in dichloromethane (15 mL). The mixture was stirred at room temperature for 5 minutes, and then directly loaded onto silica gel in a flash column. Column chromatography using a gradient (2 to 5 %) of methanol in dichloromethane afforded 101 mg (0.41 mmol, 90%) of the title compound as a purple solid. ^{1}H NMR (600 MHz, CD_4O) δ 8.51 (s, 2H), 7.57 (s, 2H), 3.61 (s, 0H), 3.03 (s, 0H). ^{13}C NMR (100 MHz, CD_4O) δ 172.39 (1 C), 168.74 (1 C), 165.19 (1 C), 141.60 (1 C), 132.19 (1 C), 131.06 (2 CH), 128.91 (2 CH), 41.65 (1 CH_2), 21.06 (1 CH_3).

1.2. Synthesis of tetrazine functionalized hyaluronic Acid (HA-Tz)

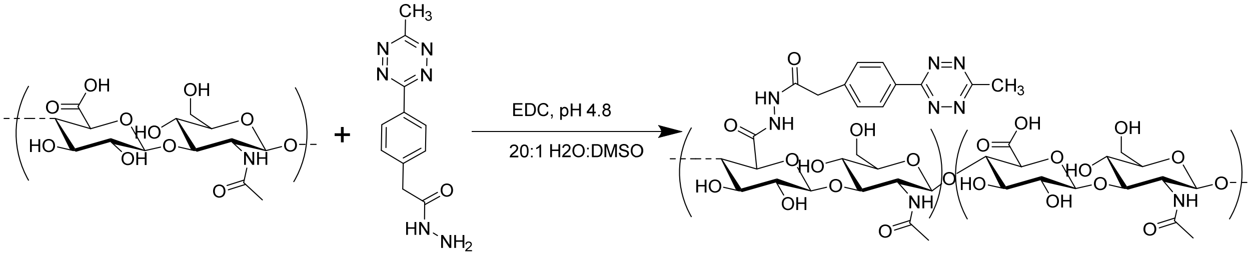

Hyaluronic acid (HA, sodium salt, 430 kDa, 275.9 mg, 0.69 mmol) was dissolved in DI H_2O (92 mL) at a concentration of 3 mg/mL. To this solution was added 1-ethyl-3-[3-(dimethylamino)propyl]-carbodiimide hydrochloride (EDC, 264.9 mg, 1.38 mmol), Tz-hydrazide (67.6 mg, 0.28 mmol) dissolved in DMSO (5 mL). The solution was then added dropwise to the HA solution. The resulting mixture was stirred at room temperature for 24 h with pH controlled around 4.8 by adding 0.1 M HCl aqueous solution. The resulting solution was diluted with DI H_2O to a final volume of 200 mL and was exhaustively dialyzed (Spectra 10 kDa MWCO) first against

0.1 M NaCl solution, then against DI H_2O. The purified solution was lyophilized to afford 267.2 mg (0.60 mmol, 88%) HA-Tz as a pink fluffy solid. The product was stored at -20 °C prior to use.

1.3. Synthesis of PEG-based TCO crosslinker (PEG-bisTCO)

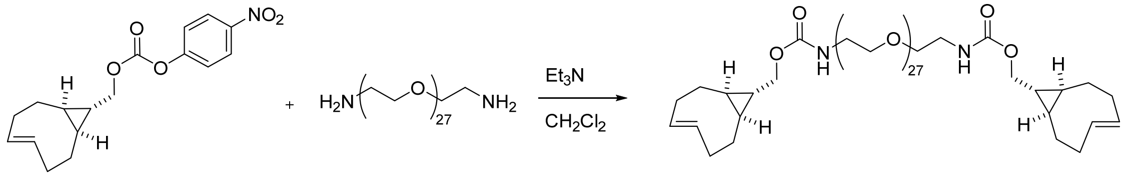

To a round bottom flask was added PEG_{27}-diamine (125 mg, 0.10 mmol), CH_2Cl_2 (2 mL) and Et_3N (56 μL, 0.40 mmol). After the addition of sTCO nitrophenyl carbonate[1] (80 mg, 0.25 mmol), the reaction mixture was stirred at room temperature overnight. Upon completion of the reaction, based on UPLC-MS analysis, the solvent was evaporated under reduced pressure and the residue was purified by flash chromatography on silica gel using 30% acetone in hexanes and then a gradient of 2-5% MeOH in CH_2Cl_2 to give the product (113 mg, 0.070 mmol, 70% yield) as water soluble semi-solid. ^{1}H NMR (600 MHz, $CDCl_3$) δ 5.83 (ddd, *J* = 16.1, 9.3, 6.2 Hz, 2H), 5.26 (s, 2H), 5.09 (ddd, *J* = 15.9, 10.2, 3.5 Hz, 2H), 3.90 (d, *J* = 6.2 Hz, 4H), 3.63 – 3.59 (m, 104H), 3.52 (t, *J* = 5.1 Hz, 4H), 3.33 (d, *J* = 5.0 Hz, 4H), 2.37 – 2.30 (m, 2H), 2.23 (m, 4H), 2.18 (m, 2H), 1.90 – 1.84 (m, 4H), 0.80 (m, 2H), 0.58 – 0.47 (m, 4H), 0.43 – 0.36 (m, 4H). ^{13}C NMR (100 MHz, $CDCl_3$) δ 156.9 (2 C), 138.4 (2 CH), 131.3 (2 CH), 70.6 (CH_2 on PEG), 70.26 (2 CH_2), 70.18 (2 CH_2), 69.41 (2 CH_2), 40.76 (2 CH_2), 38.69 (2 CH_2), 33.79 (2 CH_2), 32.60 (2 CH_2), 27.66 (2 CH_2), 24.71 (2 CH), 21.95 (2 CH), 20.93 (2 CH). LC-MS t_R= 2.48 mins, $[M+H_3O]^+$ found at 1623.90, HRMS-ESI *m/z*, ($M+2H^+/2$) calculated for $C_{78}H_{146}N_2O_{31}^{2+}$ 803.4949, found, 803.4990.

1.4. Synthesis of MMP-degradable TCO crosslinker (GIW-bisTCO)

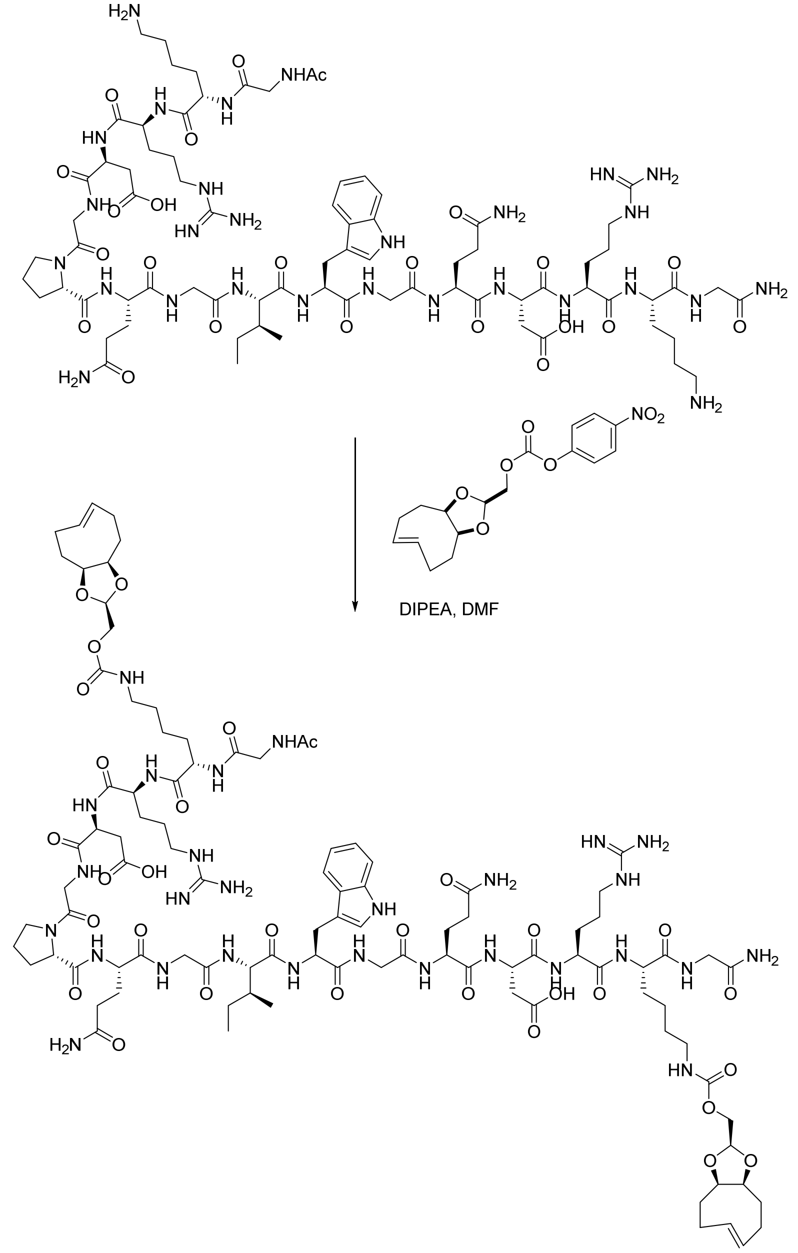

MMP-degradable peptide with a sequence of Ac-GKRDGPQGIWGQDRKG-NH_2 (abbreviated as GIW, 73 mg, 40.6 μmol), prepared by solid phase peptide synthesis, was dissolved in anhydrous DMF (1.5 mL). *N, N*-diisopropylethylamine (28 μL, 162 μmol) was added followed by dTCO-4-nitrophenyl carbonate[2] (36 mg, 102 μmol). The solution was stirred at room temperature for 2 hours. The reaction was deemed complete when only the desired bis-modification product was observed by UPLC-MS. The resulting solution was added dropwise to 35 mL of ice cold diethyl ether. Then it was centrifuged at 4000 rpm for 5 mins, and the clear ethereal solution was removed. The solid was re-dissolved in 2 mL of DMF and precipitated into ice cold diethyl ether.

The precipitation/re-dissolve cycle was repeated for a total 3 times. Analytical grade sample was obtained by purification of the white powder by reverse phase chromatography on C_{18} silica gel using a gradient of 5% to 95% MeOH in neutral water. LC-MS for GIW-bisTCO: t_R = 1.72 mins, $[M+2H]^{2+}$ found 1109.21. HRMS-ESI for GIW-bisTCO: *m/z*, ($M+2H^+$ /2) calculated for $C_{98}H_{153}N_{28}O_{31}^{2+}$ 1109.0623, found, 1109.0621. LC-MS for GIW: t_R = 1.10 mins, $[M+4H]^{4+}$ found 449.94. HRMS-ESI for GIW, *m/z*, ($M+2H^+$ /2) calculated for $C_{76}H_{125}N_{28}O_{23}^{2+}$ 898.9731, found, 898.9726.

1.5. Synthesis of PEG-TCO

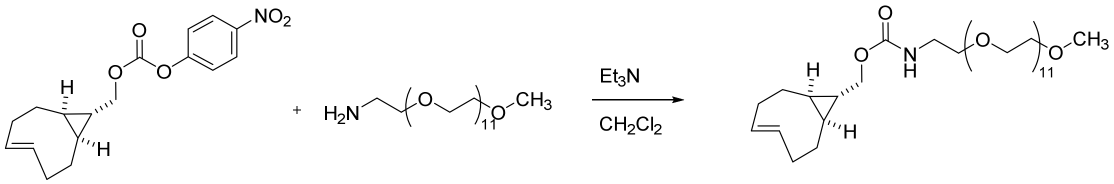

To a round bottom flask was added the MeO-PEG_{12}-amine (67 mg, 98% purity, 0.116mmol), CH_2Cl_2 (2 mL) and Et_3N (65µL, 0.464mmol). After the addition of sTCO 4-nitrophenyl carbonate (55 mg, 0.175 mmol), the solution was stirred at room temperature overnight, at which point the reaction was analyzed for completion by UPLC-MS analysis. The solvent was evaporated under reduced pressure and the residue was passed through a short column of deactivated C_2 silica gel first eluting with CH_2Cl_2 to remove the excess carbonate and then 10% MeOH in CH_2Cl_2 to elute fractions containing the product. The solvent was concentrated under reduced pressure and the yellow crude product was purified by reverse phase chromatography using a Biotageon C_{18} silica gel using a gradient of 10% to 90% MeOH in neutral water to give the product (63 mg, 0.085 mmol, 74% yield) as a pale yellow oil. ^{1}H NMR (600 MHz, $CDCl_3$) δ 5.83 (ddd, *J* = 16.1, 9.3, 6.2 Hz, 1H), 5.23 (s, 1H), 5.09 (ddd, *J* = 15.9, 10.5, 3.1 Hz, 1H), 3.91 (d, *J* = 6.1 Hz, 2H), 3.66 – 3.58 (m, 42H), 3.55 – 3.49 (m, 4H), 3.35 (s, 3H), 3.33 (d, *J* = 5.1 Hz, 2H), 2.38 – 2.29 (m, 1H), 2.28 – 2.20 (m, 2H), 2.18 (dt, *J* = 12.7, 8.2 Hz, 1H), 1.93 – 1.84 (m, 2H), 0.80 (td, *J* = 12.1, 7.1 Hz, 1H), 0.55 – 0.46 (m, 2H), 0.42 – 0.34 (m, 2H). ^{13}C NMR (100 MHz, $CDCl_3$) δ 156.9 (C), 138.4 (CH), 131.3 (CH), 71.9 (CH_2), 70.60 (CH_2 on PEG), 70.56 (CH_2 on PEG), 70.52 (CH_2 on PEG), 70.3 (CH_2), 70.2 (CH_2), 69.4 (CH_2), 59.1 (CH_3), 40.8 (CH_2), 38.7 (CH_2), 33.8 (CH_2), 32.6 (CH_2), 27.7 (CH_2), 24.70 (CH), 21.95 (CH), 20.93 (CH). LC-MS t_R = 2.11 mins, $[M+NH_4]^+$ found at 755.71, HRMS-ESI *m/z*, ($M+H^+$) calculated for $C_{36}H_{68}NO_{14}$ 738.4634, found, 738.4651.

1.6. Synthesis of RGD-TCO

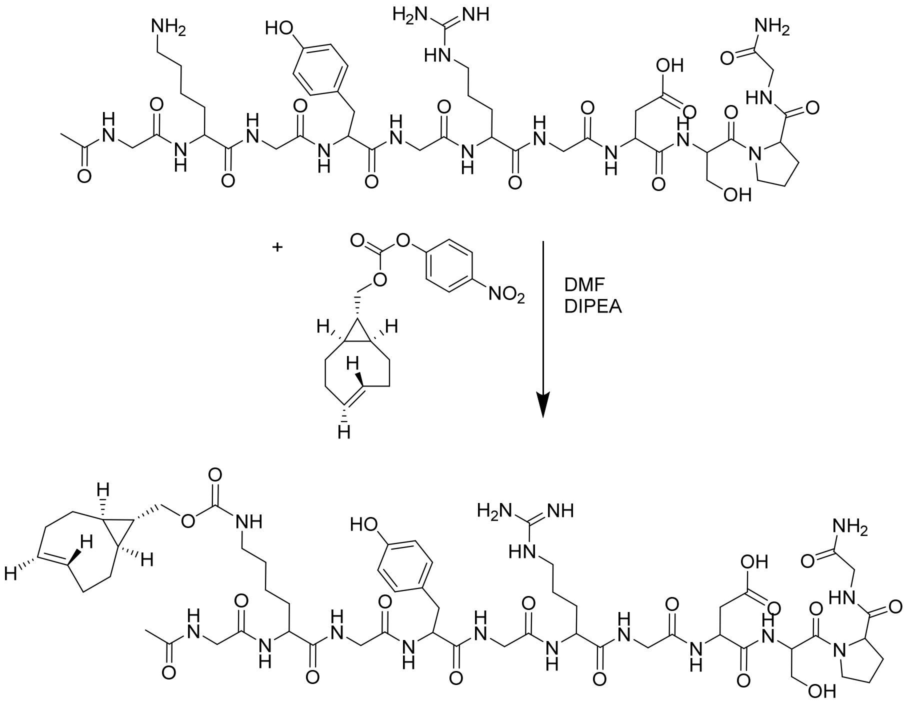

Prior to TCO conjugation, RGD peptide with a sequence of GKGYGRGDSPG was synthesized following standard solid phase peptide synthesis protocol. The cleaved product, with C-amidated and N-acetylated, was allowed to react with nitrophenyl carbonate-derived sTCO in anhydrous DMF to install TCO through the lysine amine. The product was purified by HPLC and analyzed by ESI-MS, as reported in our previous publication.[3]

1.7. Synthesis of PEG-dTCO

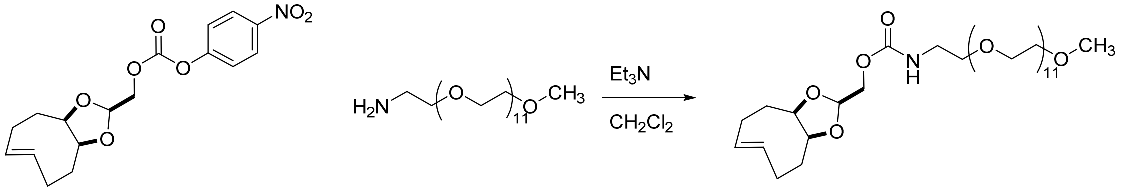

To a round bottom flask was added the MeO-PEG_{12}-amine (58 mg, 98% purity, 0.102mmol), CH_2Cl_2 (2 mL) and Et_3N (56μL, 0.400mmol). Next, dTCO 4-nitrophenyl carbonate (53mg, 0.152mmol, 91:9 ratio of 2 diastereomers) was added and the solution was stirred at room temperature overnight. When the reaction was complete, as judged by UPLC-MS analysis, the solvent was evaporated under reduced pressure and the residue was passed through a short C_2 deactivated silica gel column first eluting with CH_2Cl_2 to remove the excess carbonate and then 10% MeOH in CH_2Cl_2 to get fractions containing the product. The solvent was concentrated under reduced pressure and the yellow crude product was purified by reverse phase chromatography

on C_{18} silica gel using a gradient of 10% to 90% MeOH in neutral water to give the product (60 mg, 0.078 mmol, 76% yield) as a pale yellow oil. ^{1}H NMR (400 MHz, $CDCl_3$) δ 5.64 – 5.54 (m, 1H), 5.54 – 5.43 (m, 1H), 5.30 (s, 1H), 4.89 (m, 1H), 4.15 – 4.01 (m, 2H), 3.99 – 3.86 (m, 2H), 3.64 – 3.55 (m, 42H), 3.51 (q, J = 5.2, 4.5 Hz, 4H), 3.35 (s, 3H), 3.34 – 3.23 (m, 2H), 2.38 (m, 1H), 2.20 (m, 2H), 2.13 – 2.02 (m, 1H), 1.85 (m, 1H), 1.78 (m, 1H), 1.74 – 1.59 (m, 1H), 1.59 – 1.42 (m, 1H). ^{13}C NMR (100 MHz, $CDCl_3$) δ 156.0 (C), 136.5 (CH), 131.1 (CH), 99.1 (CH), 82.8 (CH), 80.7 (CH), 71.9 (CH_2), 70.6 (CH_2 on PEG), 70.6 (CH_2 on PEG), 70.5 (CH_2 on PEG), 70.3 (CH_2), 70.0 (CH_2), 65.2 (CH_2), 59.1 (CH_3), 40.9 (CH_2), 38.7 (CH_2), 33.6 (CH_2), 25.5 (CH_2). LC-MS t_R = 1.89 mins, $[M+NH_4]^+$ found at 787.67. HRMS-ESI *m/z*, $(M+H^+)$ calculated for $C_{36}H_{68}NO_{16}$ 770.4533, found, 770.4550.

1.8. Synthesis of Alexa-TCO

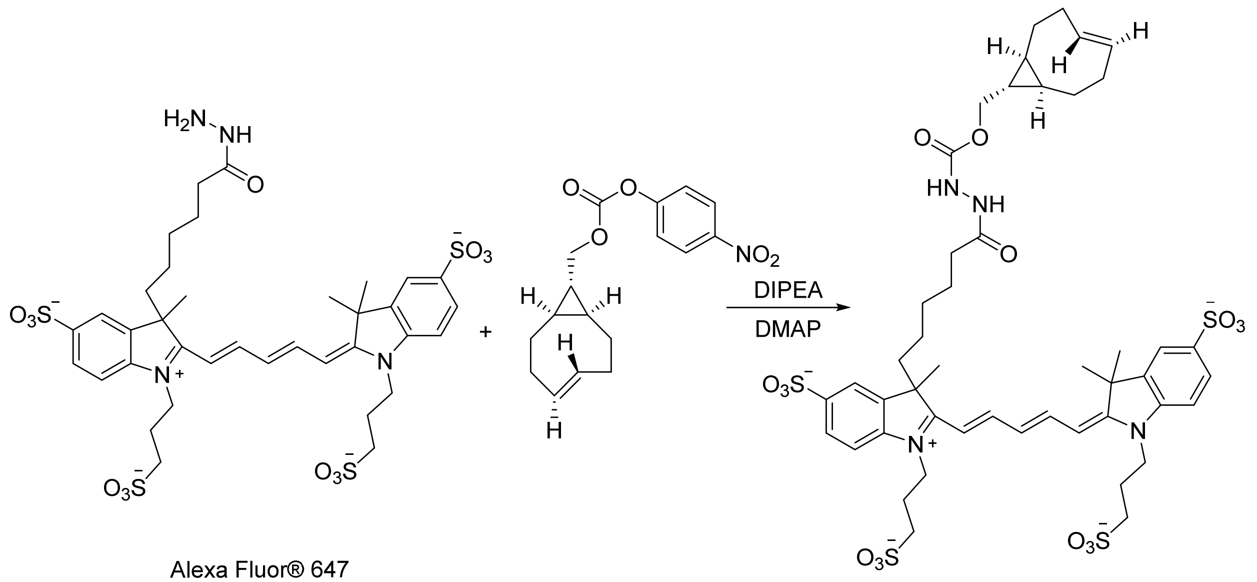

Alexa Fluor® 647

(1*R*,8*S*,9*R*,4*E*)-Bicyclo[6.1.0]non-4-en-9-ylmethyl(4-nitrophenyl) carbonate (1.3 mg, 4.2 μmol) was added to a vial that contained Alexa Fluor® 647 hydrazide, tris(triethylammonium) salt (1.0 mg, 0.83 μmol). A DMF solution (200 μL, anhydrous) containing *N,N*-diisopropylethylamine (215 μg, 1.67 μmol) and 4-dimethylaminopyridine (DMAP, 50 μg, 0.41 μmol) was added to the vial. The mixture was stirred overnight at ambient temperature and was purified with reverse phase HPLC, generating 0.49 mg (47 μmol, 56%) of Alexa-TCO as a blue solid. LC-MS and HPLC analyses indicated that the purity of a compound with a mass of 1047 Da. was >98%, as reported in our previous publication.[4]

2. Analytical methods.

***2.1. Percent tetrazine incorporation in HA-Tz*.** The percent tetrazine incorporation in HA-Tz was determined collectively by UV-vis and ^{1}H NMR analyses. UV-vis quantification was based on the tetrazine absorption at λ_{max} 267 nm, employing Beer-Lambert law. Using an aqueous solution of Tz-hydrazide at a concentrations from 4.7 mM to 0.47 mM as the standard (Figure S1A-B), the molar extinction coefficient of the tetrazine moiety (ε_{Tz}) was determined as 2.3×10^{4} L Mol^{-1} cm^{-1}. Taking into consideration the change of the molecular weight for HA disaccharide repeats after tetrazine incorporation, the degree of tetrazine incorporation was calculated as 18.6% (Figure S1C). By ^{1}H NMR (Figure S11), tetrazine incorporation in HA was calculated as 18.0%, analyzed by comparing the integration between the aromatic protons (7.4-8.4 ppm) to the anomeric protons of HA. As expected, some EDC-activated carboxyl groups in HA were transformed to N-acylurea.[5]

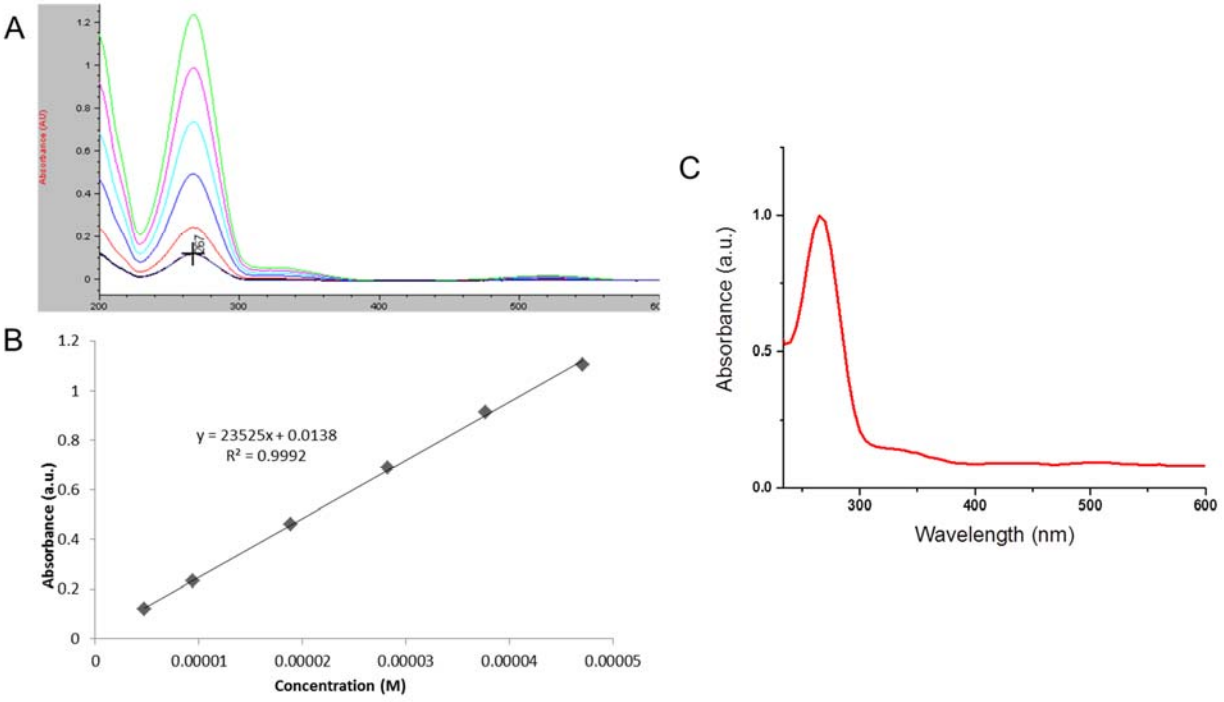

Figure S1. UV-Vis spectra of aqueous solutions of Tz-hydrazide (A, 4.7 mM to 0.47 mM) and HA-Tz (C, 0.27mM). The extinction coefficient was determined from the standard curve with a linear regression (B). A UV cuvette with a pathlength of 1 cm was used.

2.2. Analysis of reaction kinetics.

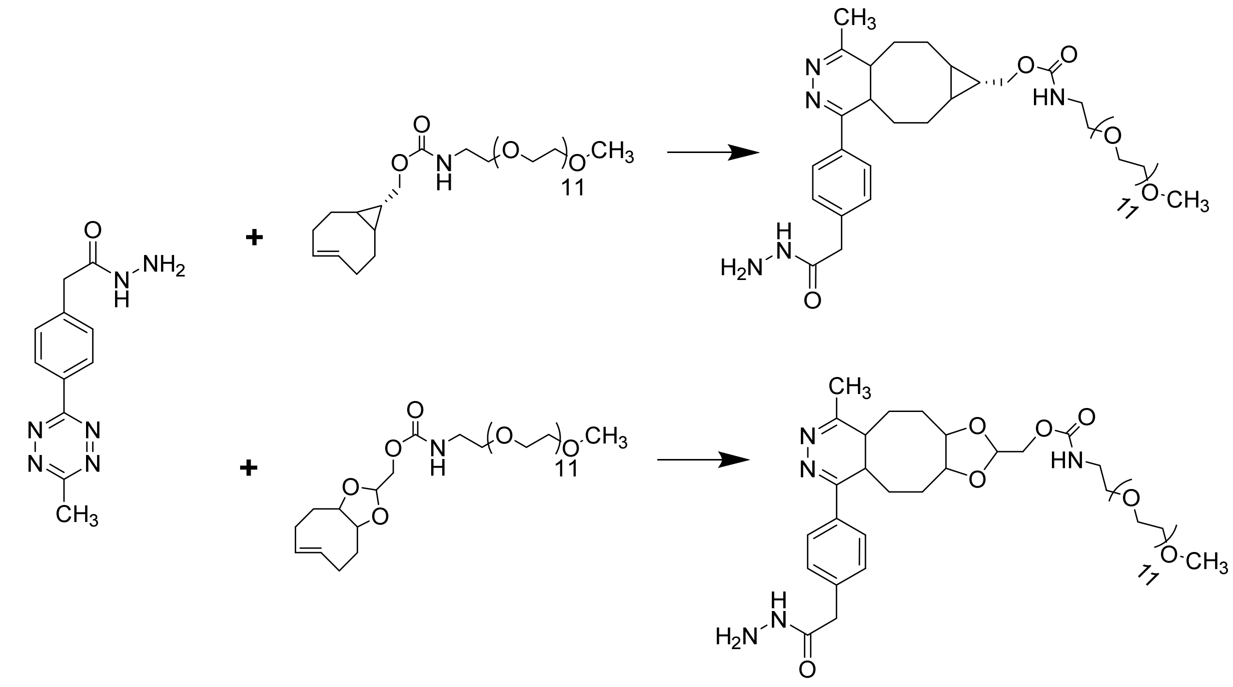

The reaction was run under pseudo-first order conditions and monitored by UV-Vis spectroscopy at 267 nm using an Applied Photophysics SX.18MV-R stopped-flow dual mixing spectrometer. The reactants were separately dissolved in H_2O and mixed in the stopped-flow device. The initial concentrations of Tz-hydrazide, PEG-TCO and PEG-dTCO were 4.43×10^{-5} M, 4.02×10^{-4} M and 4.28×10^{-4} M, respectively, with a 1:1 volume mixing. The spectrum was acquired every 5×10^{-4} seconds for 1 second and 2.5×10^{-3} for 5 seconds for PEG-TCO and PEG-dTCO, respectively. The K_{obs} was determined by fitting a non-linear curve of ln(A/A_0) vs time, where A_0 and A was absorbance at time 0 and t, respectively. The kinetic runs were measured in triplicate, and the average K_{obs} was 13.51 ± 0.01 s^{-1} and 2.130 ± 0.003 s^{-1} for PEG-TCO and PEG-dTCO, respectively. The second order rate constant (k_2) was calculated to be 6.70×10^4 $M^{-1}s^{-1}$ and 9.94×10^3 $M^{-1}s^{-1}$ for PEG-TCO and PEG-dTCO**,** respectively.

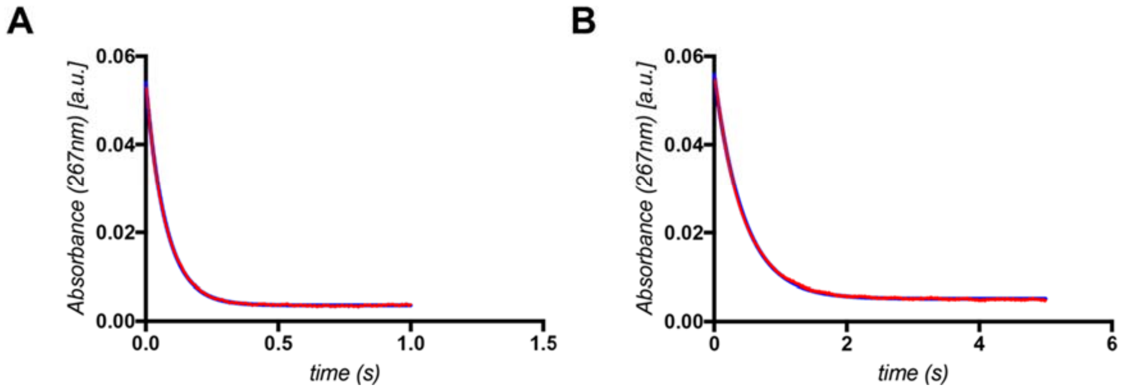

Figure S2. Stopped flow results for reactions of Tz-hydrazide with PEG-TCO (**A**) or with PEG-dTCO (**B**). Red circles: raw data; Blue line: fitted curve.

2.3. Mechanical Properties. Hydrogel microspheres were tested under compression using a micro-materials tester, with a parallel plate (Video S1). The upper platen was positioned ~150 µm above the hydrogel sphere. Following hydration by a single drop of PBS the platen was driven toward the hydrogel microsphere at 45 µm/s to a target depth of 700 µm. Due to the known load cell compliance (0.0341 mN/µm) and surface offset (~150 µm), the compression rate and deformation of the sample were typically ~40 µm/s and ~500 µm, respectively. Preliminary experiments demonstrated that the compression response was relatively insensitive (± 0.7 kPa or ± 4.7%) to compression rates between 5 and 400 µm/s. From Figure S3, it can be seen that as the glass flat approaches the hydrogel microsphere, the force remains at zero until a critical point at which the formation of a fluid meniscus pulls the cantilever beam of the load cell. We define the point of initial contact as the point during which the meniscus is first formed. As the glass platen continues downward the force increases nonlinearly with the compression of the sphere in accordance with Hertz's theory for the contact between spherical elastic bodies. A Hertzian analysis of the loading portion of the test was used to quantify the compressive modulus of each gel. Hertz's solution to the deformation of an elastic sphere against a rigid flat is: $E_c = \frac{3}{4} \cdot F \cdot R^{-\frac{1}{2}} \cdot \left(\frac{\delta}{2}\right)^{-\frac{3}{2}}$. The model determines the contact modulus (E_c) of the sphere based on the measured variables: force (F), radius of the sphere (R), and deformation (δ). The analysis assumes the glass is rigid relative to the hydrogel microspheres and has infinite curvature, i.e. flat. Young's modulus (E) can be calculated from the contact modulus through the relationship: $E = E_c \cdot (1 - \nu^2)$, which requires prior knowledge of the Poisson's ratio (ν). Here we assume ν = 0.5. Note that the R of each sphere was measured using calipers prior to compression testing

and was paired with its indentation profile for model fitting. It is worth noting that between each indentation, the sample was removed and placed back in PBS while the surfaces were wiped free of any liquid.

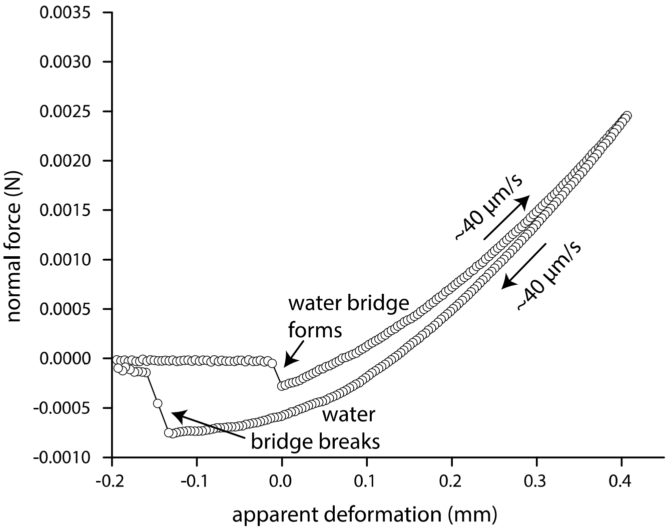

Figure S3. Representative data set demonstrating the full approach and retraction phases. Note that the formation and breaking of the fluid meniscus do not occur at the same position. Furthermore, note the apparent hysteresis between the approach and retraction curve. It is thought that the majority of the hysteresis seen here is due to the meniscus forces.

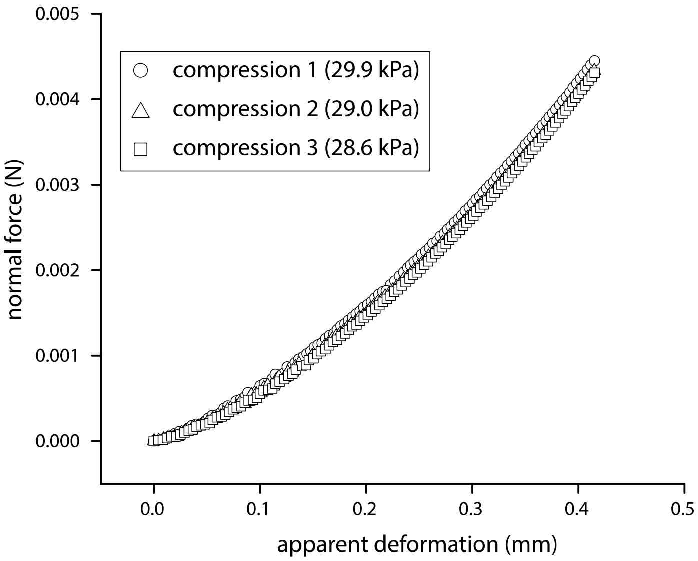

Figure S4. Repeat ramp compression experiments on a single gel. Note that in between each compression, the gel was removed and submerged in solution while the compression platens were wiped free of fluid. The standard deviation between the 3 consecutive tests was ± 0.8 kPa or ± 2.7%.

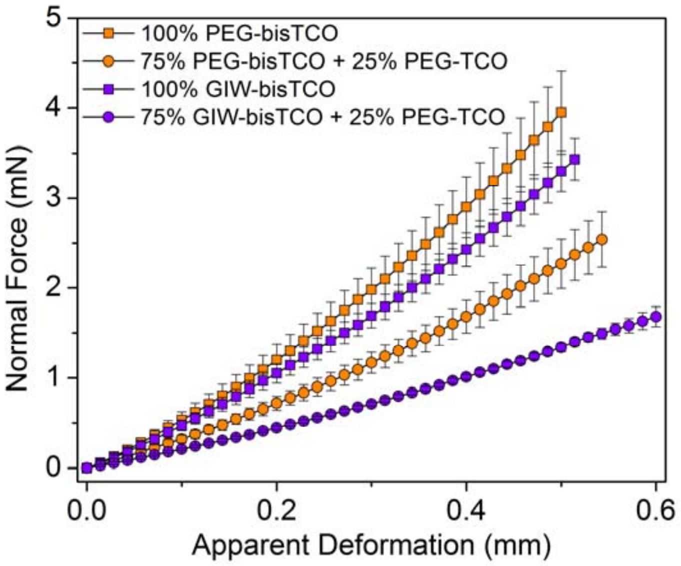

Figure S5. Force deformation curves for hydrogels crosslinked with either PEG-bisTCO or GIW-bisTCO. Modulus was altered by tuning the relative concentration of mono-functional capper, PEG-TCO.

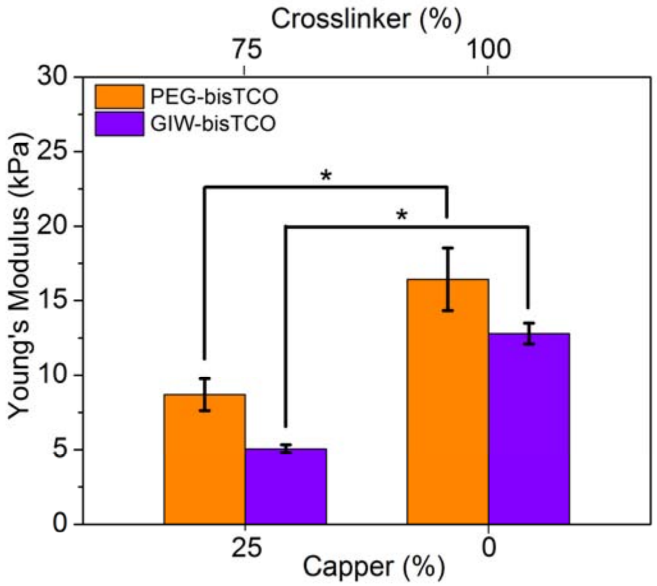

Figure S6. Young's modulus of hydrogels crosslinked with either PEG-bisTCO or GIW-bisTCO. Modulus was altered by tuning the relative concentration of mono-functional capper, PEG-TCO. * $p < 0.05$.

2.4. Gel degradation and cell morphology

As-synthesized, fully swollen hydrogel spheres were introduced to glass cylinders of known weights and the initial gel mass was recorded. Hank's balanced salt solution (HBSS, pH 7.4, 200 μL) containing 100 U/mL collagenase type IV was introduced to the cylinders and enzymatic degradation was monitored every 30 min for up to 4 h. At each time point, the buffer was aspirated and the combined weight of the glass cylinder and the gel was measured. Three repeats for each condition were included and the results were reported as the average gel mass remaining as a function of incubation time.

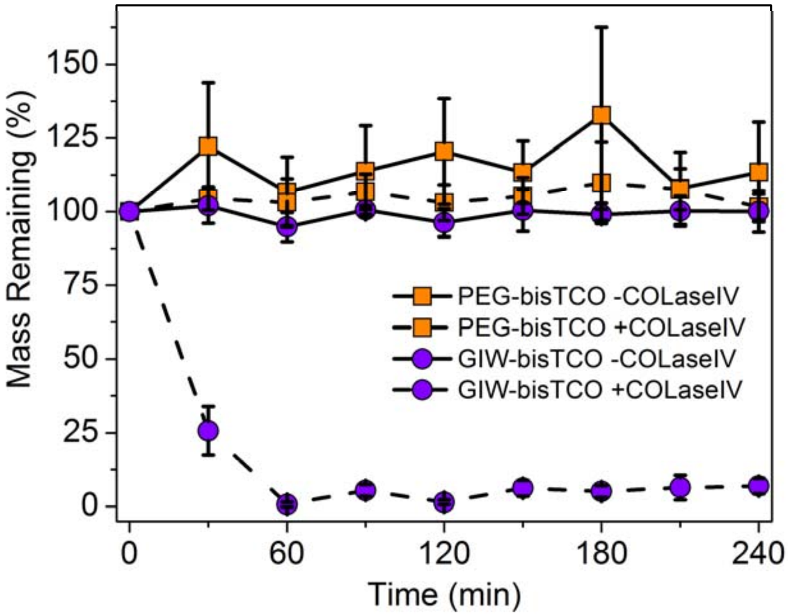

Figure S7. Hydrogel degradation. HA-Tz was crosslinked by either PEG-bisTCO or GIW-bisTCO. Hydrogels were incubated with or without of collagenase type IV and the gel mass was measured every 30 min for 4 h.

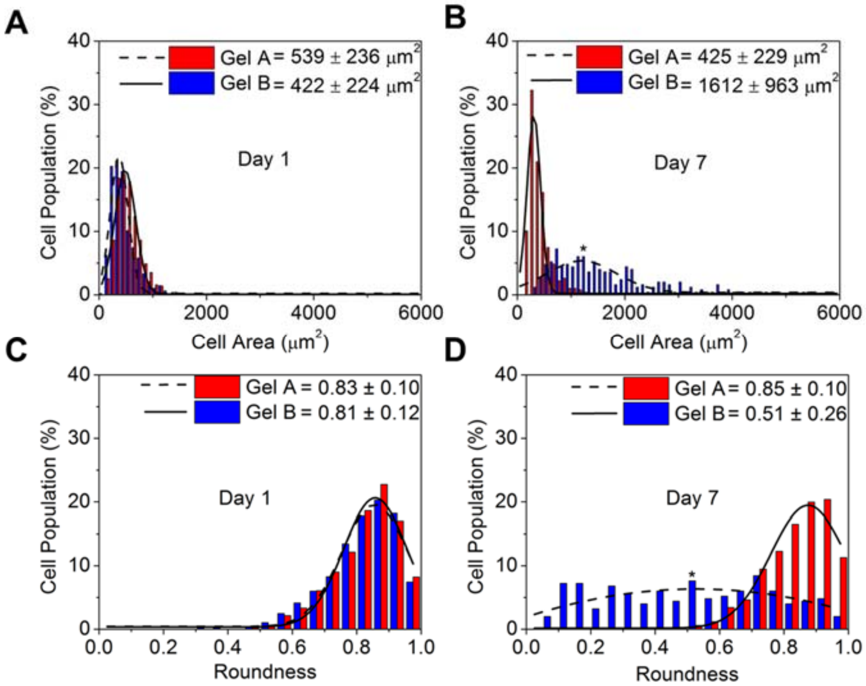

Figure S8. Characterization of cell morphology as a function of gel composition and culture time. hMSCs were dispersed in Gel A (red) or Gel B (blue). Cultures were maintained for 1 (A, C) and 7 (B, D) days. Cell area (A-B) and roundness (C-D) were assessed from the confocal images of the immunostained constructs, shown in Figure 3B.

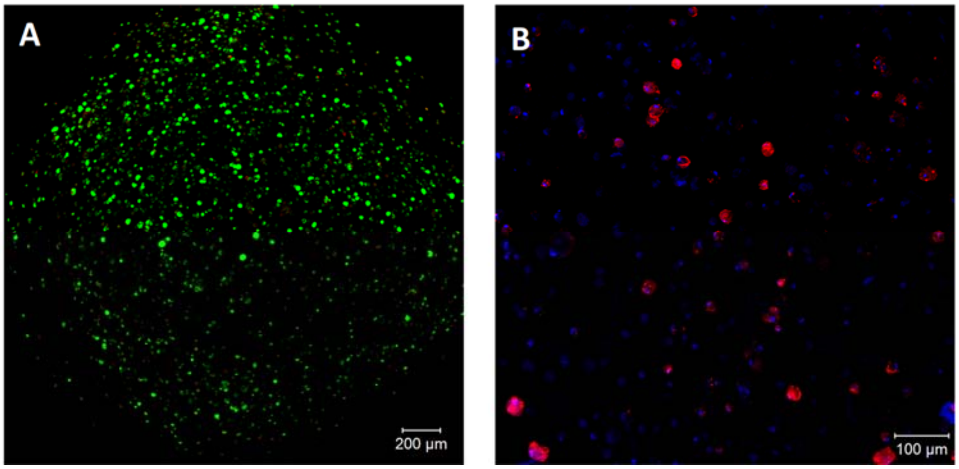

Figure S9. 3D culture of hMSCs in a homogeneous hydrogel prepared using HA-Tz (5%), PEG-bisTCO (1.77 mM) and RGD-TCO (0.19 mM). (A) Representative confocal image of the cellular construct stained by calcein AM (green) and ethidium homodimer (red) for live and dead cells, respectively, after 7 days of culture. (B) Representative confocal image of the cellular construct stained by F-actin (red) and DAPI (blue) after 7 days of culture.

2.5. Immunostaining for collagen I.

After 7 days of culture, constructs were stained for F-actin using Alexa Fluor 568 phalloidin, with the nuclei counter stained by DAPI, following our previous protocols.[6] Samples were incubated with primary anti-Collagen I antibody (Abcam) at a 1:100 dilution in 1x PBS containing 3% BSA for 2 h at room temperature. Samples were then treated with Alexa Fluor 488-conjugated secondary antibody at a 1:200 dilution in the same buffer for 2 h at room temperature. Stained samples were imaged using a Zeiss 710 NLO confocal microscope with a 40X objective.

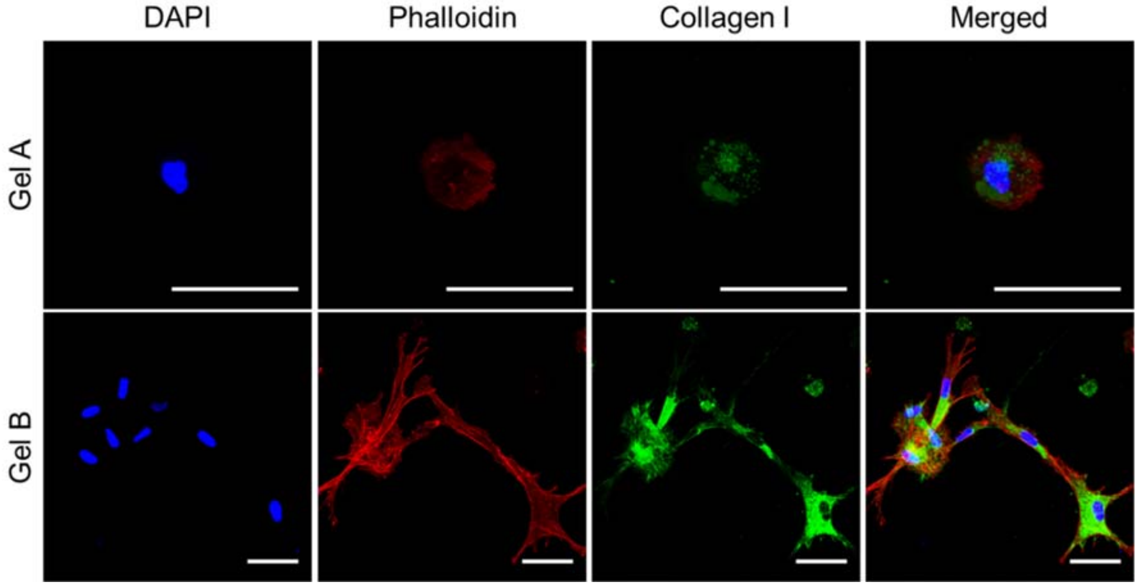

Figure S10. Characterization of cellular expression of collagen I by immunostaining and confocal imaging (40X). Cells were cultured for 7 days in homogeneous hydrogels prepared using either PEG-bisTCO (Gel A) or GIW-bisTCO and RGD-TCO (Gel B). DAPI, Phalloidin and Collagen I were stained blue, red and green, respectively. Scale bar: 50 μm.

3. Raw NMR and MS spectra (Figure S11-S34)

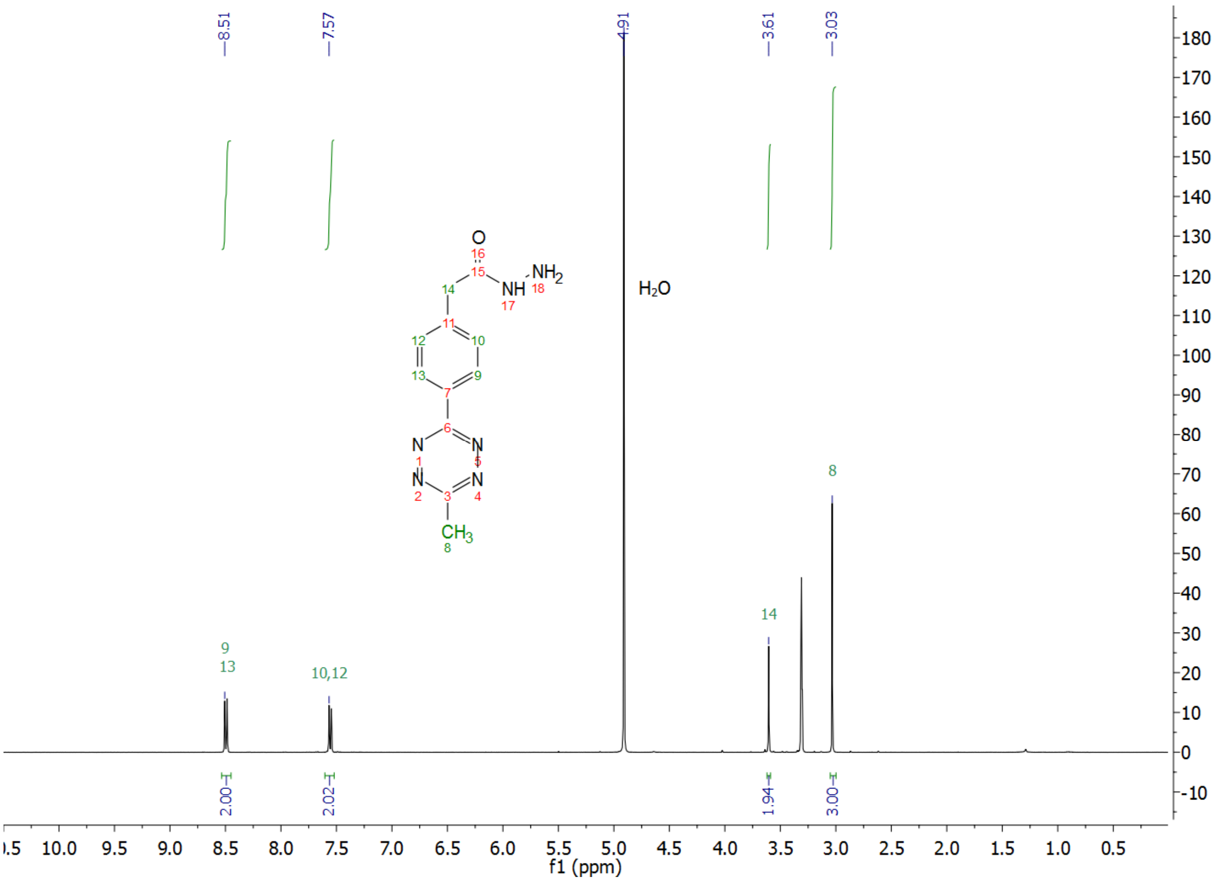

Figure S11. ^{1}H NMR spectrum of Tz-hydrazide in methanol-d_4.

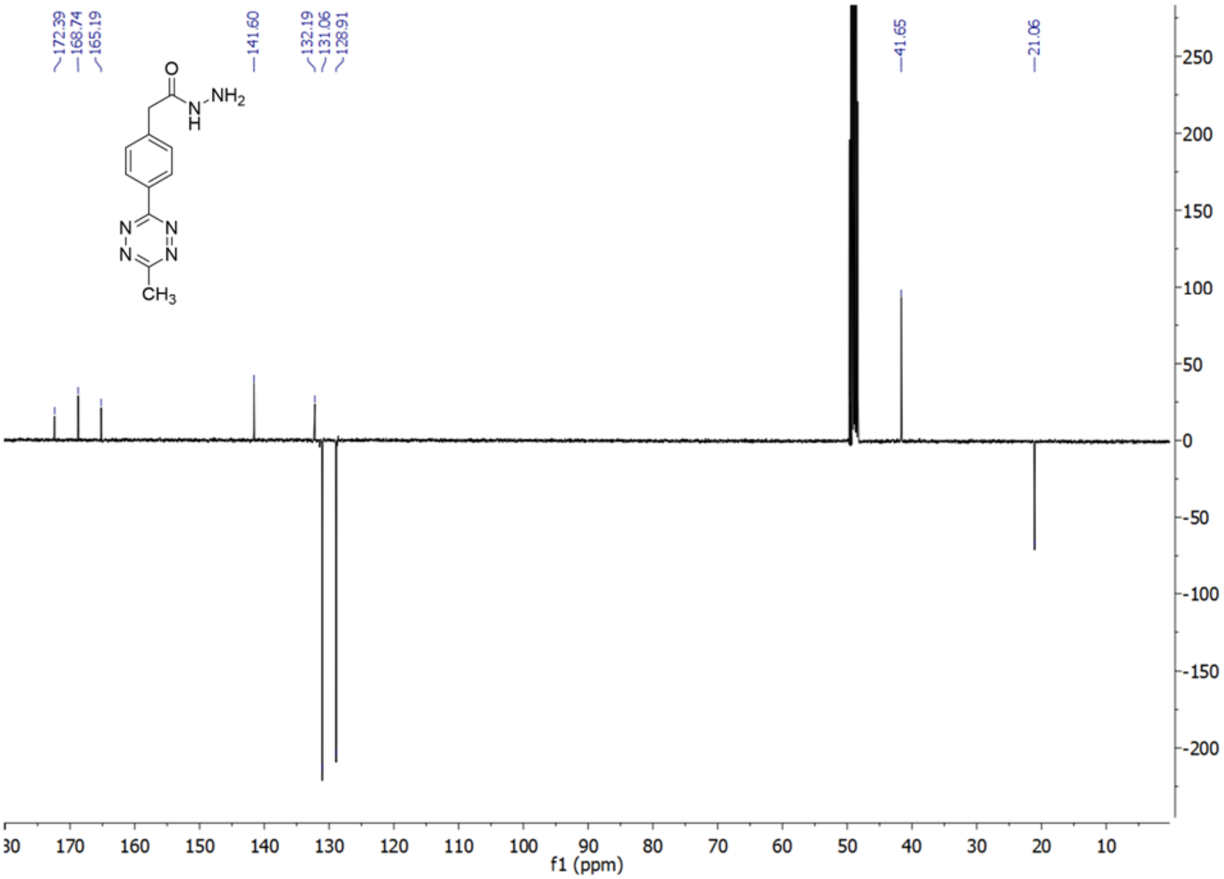

Figure S12. ^{13}C NMR spectrum of Tz-hydrazide in $CDCl_3$

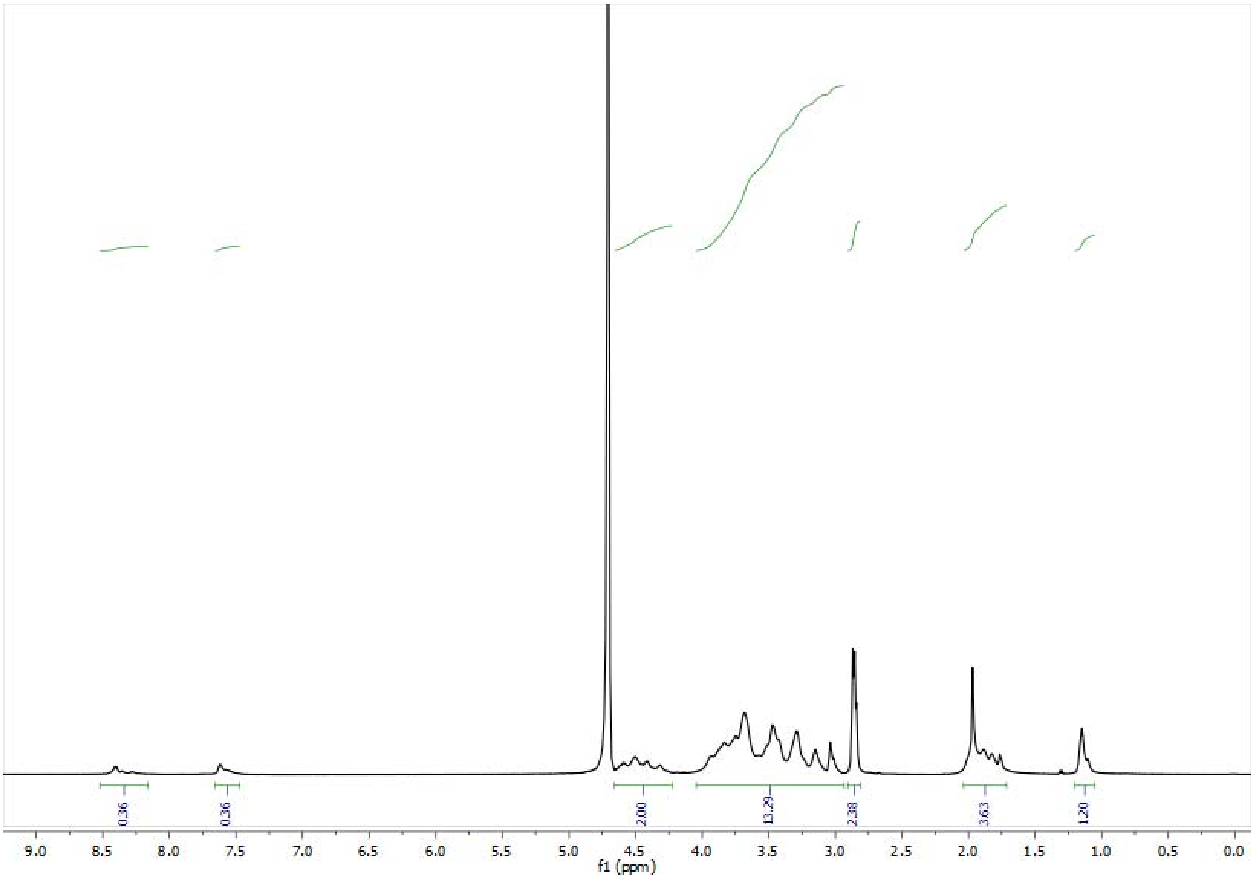

Figure S13. ^{1}H NMR spectrum of HA-Tz in D_2O.

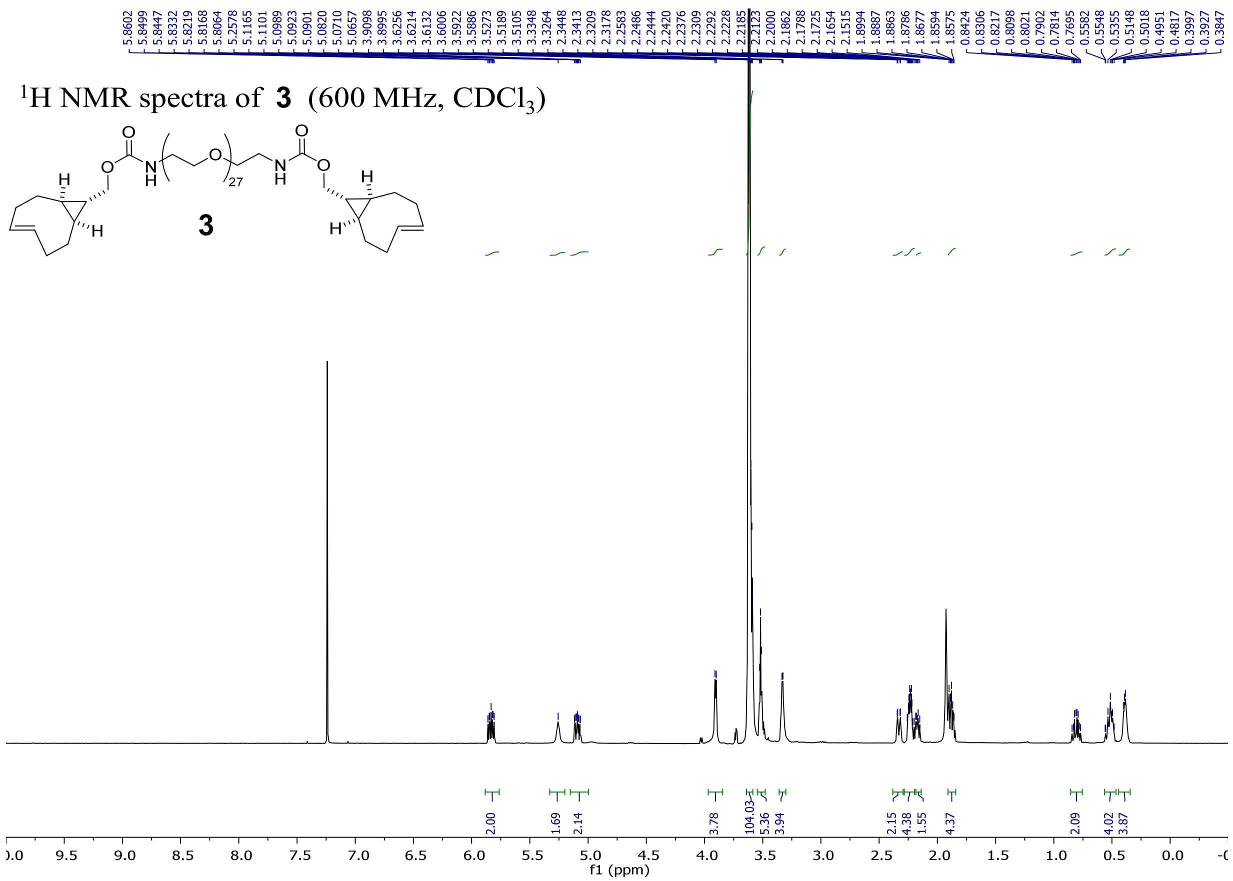

Figure S14. ^{1}H NMR spectrum of PEG-bisTCO in $CDCl_3$

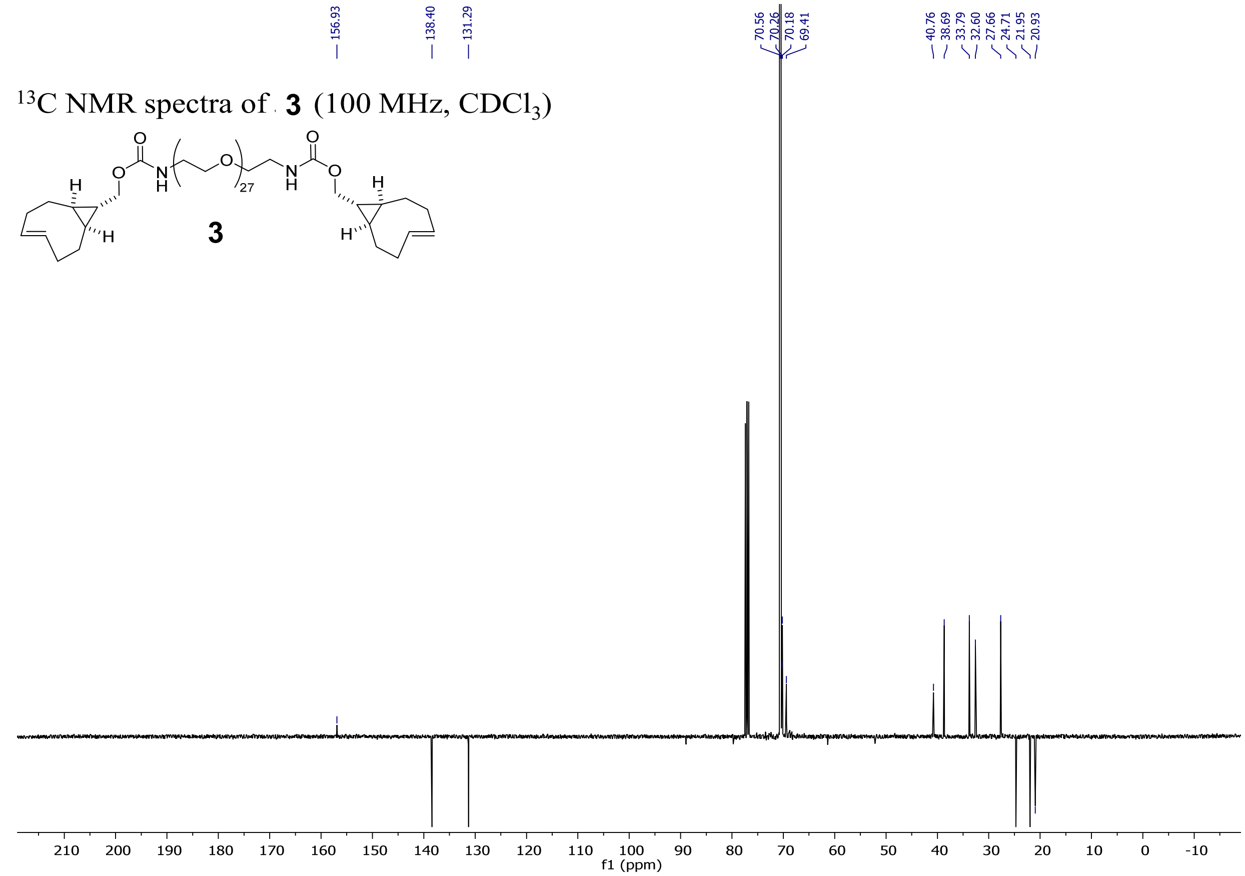

Figure S15. ^{13}C NMR spectrum of PEG-bisTCO in $CDCl_3$

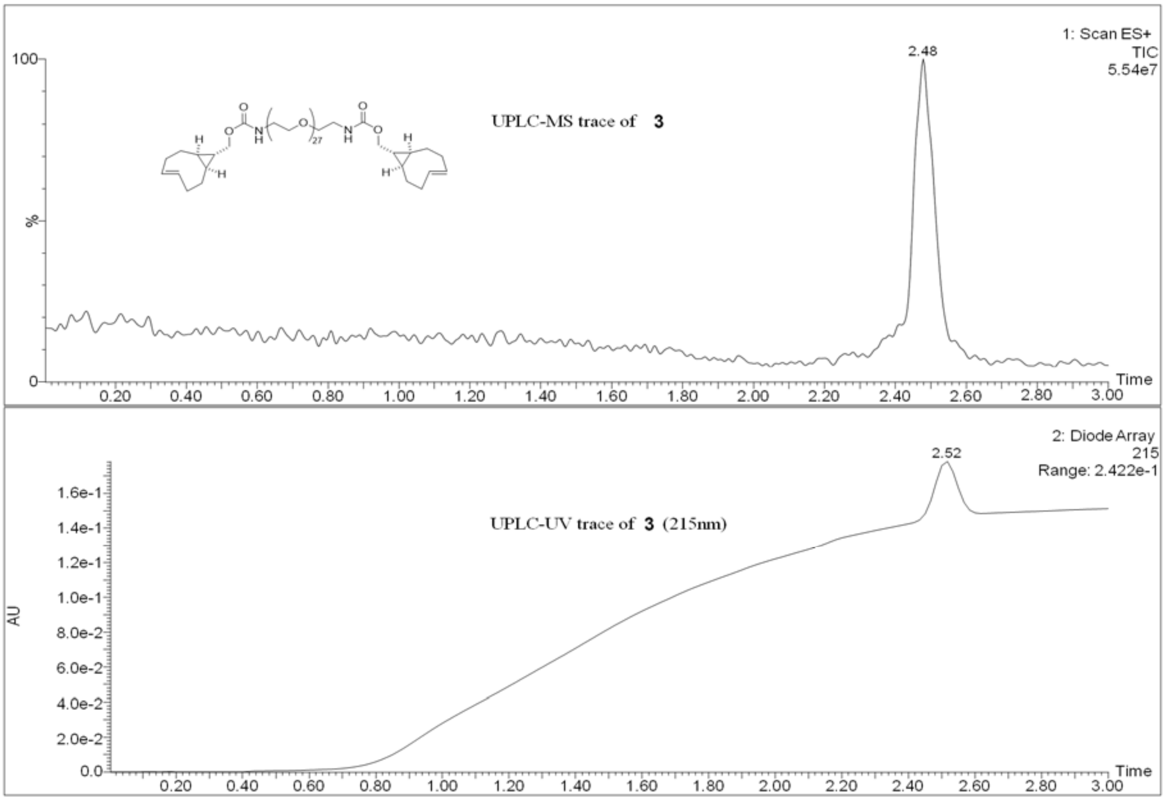

Figure S16. UPLC-MS trace of PEG-bisTCO.

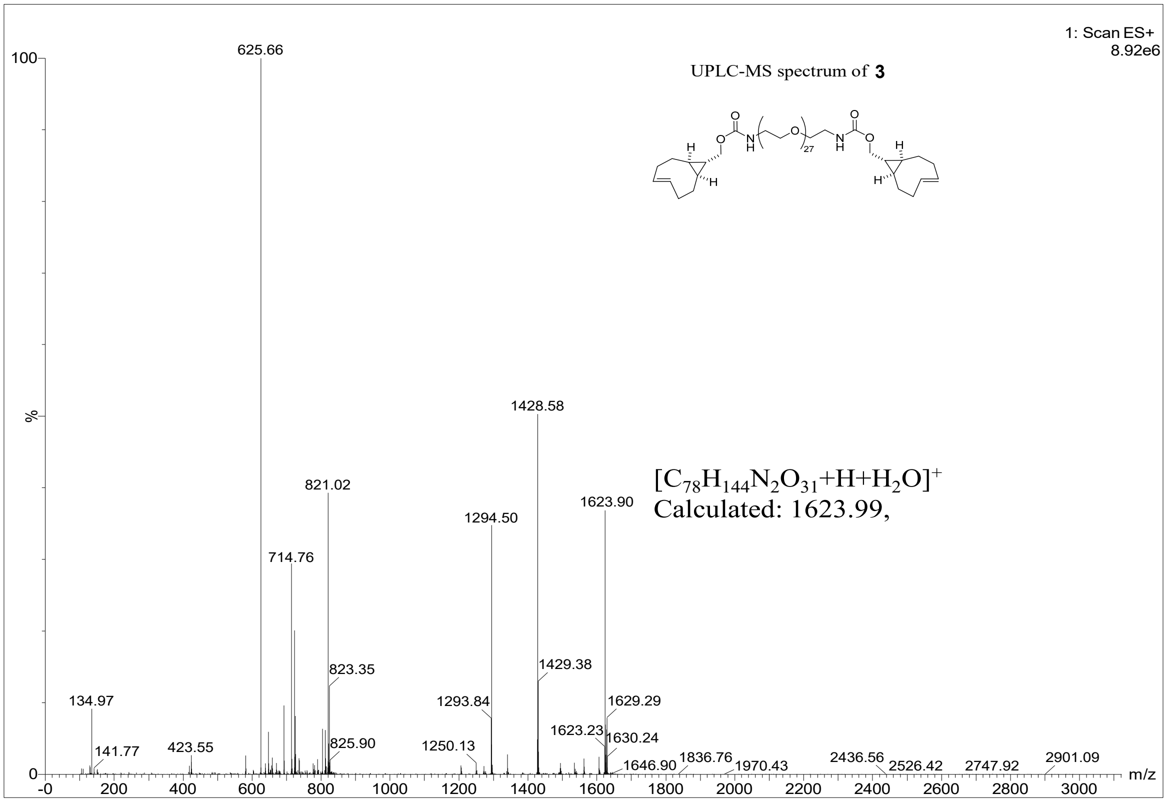

Figure S17. UPLC-MS spectrum of PEG-bisTCO.

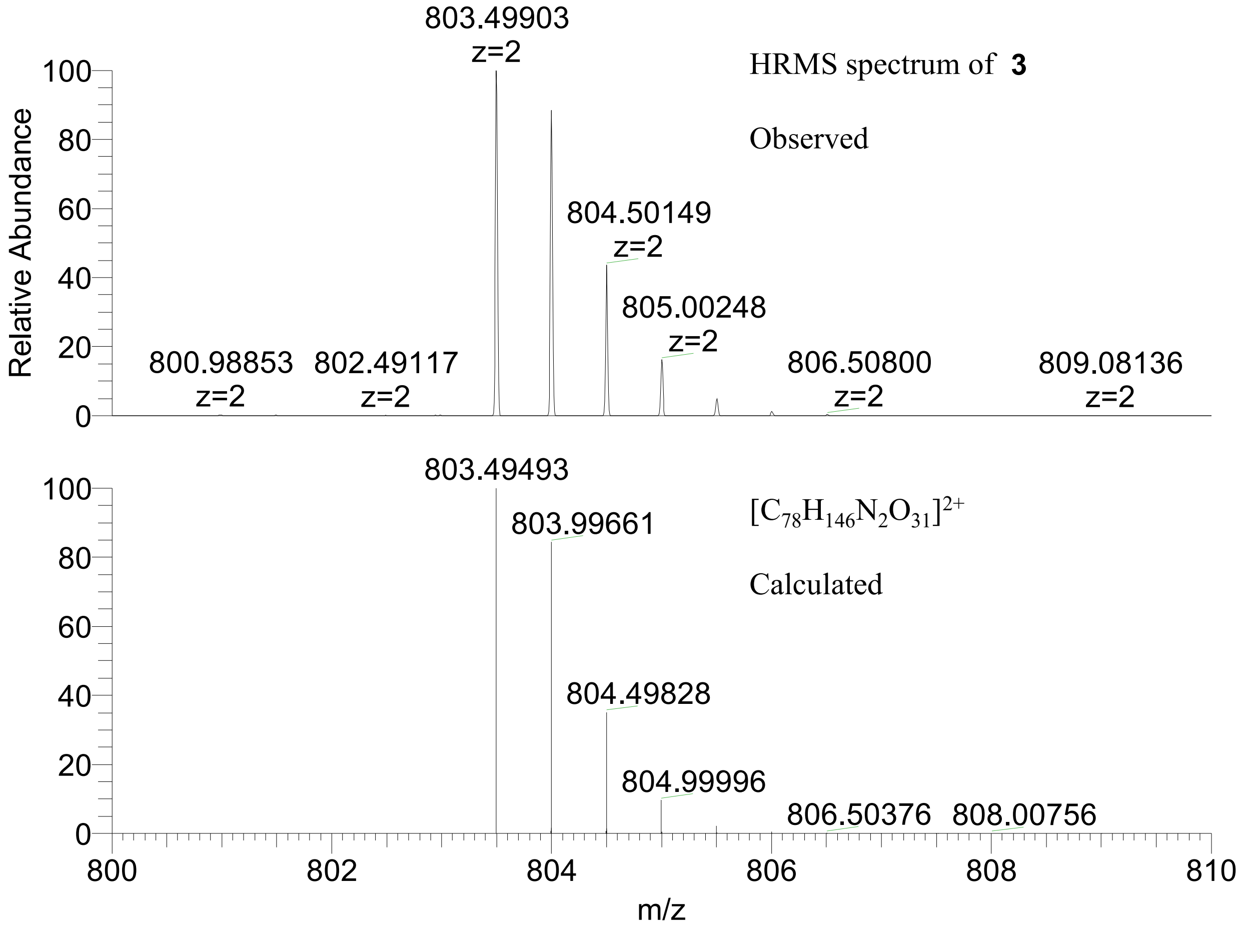

Figure S18. HRMS spectrum of PEG-bisTCO.

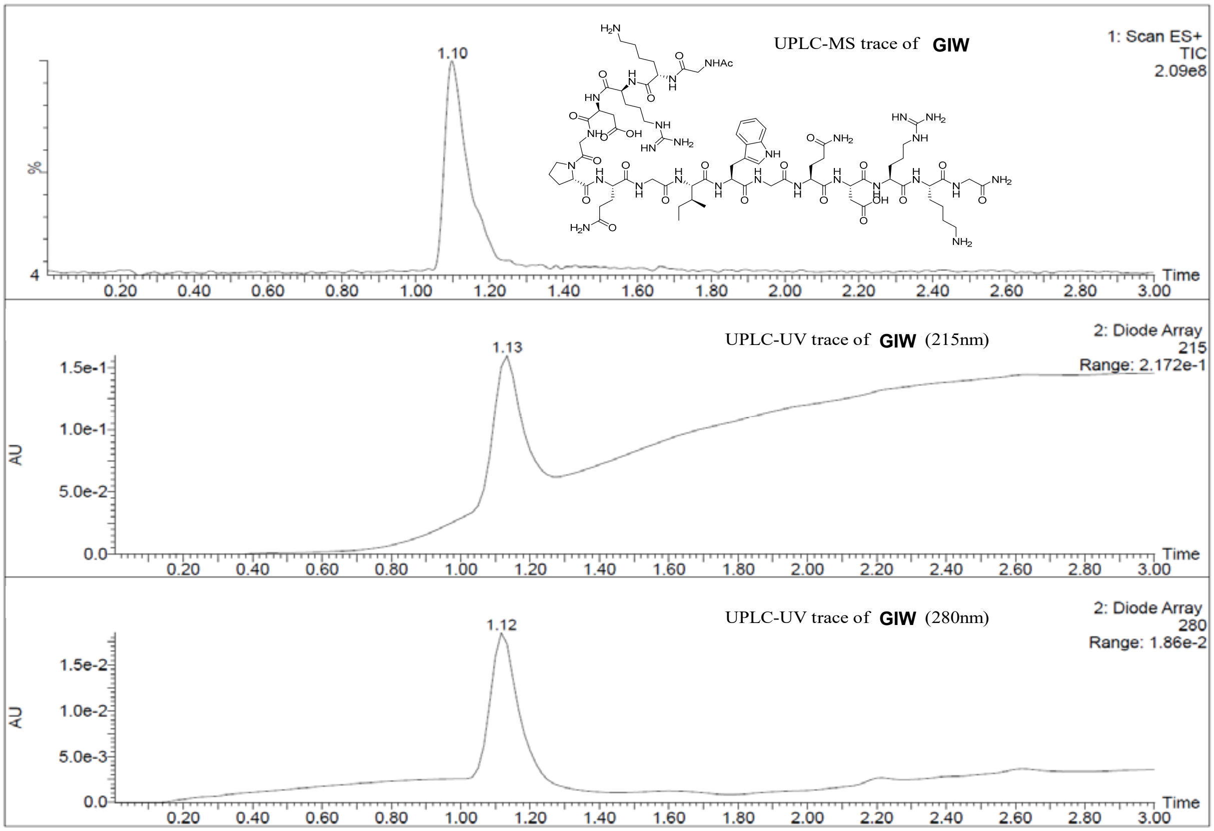

Figure S19. UPLC-MS trace of the GIW peptide.

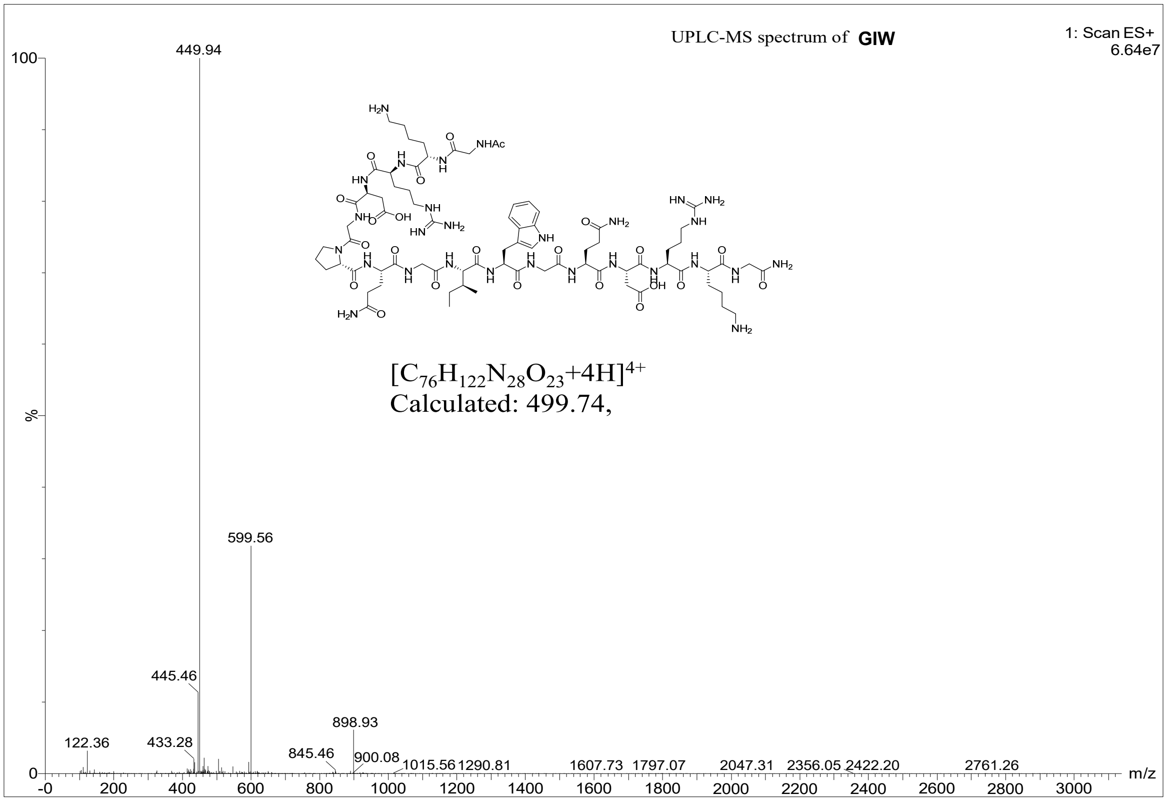

Figure S20. UPLC-MS spectrum of the GIW peptide.

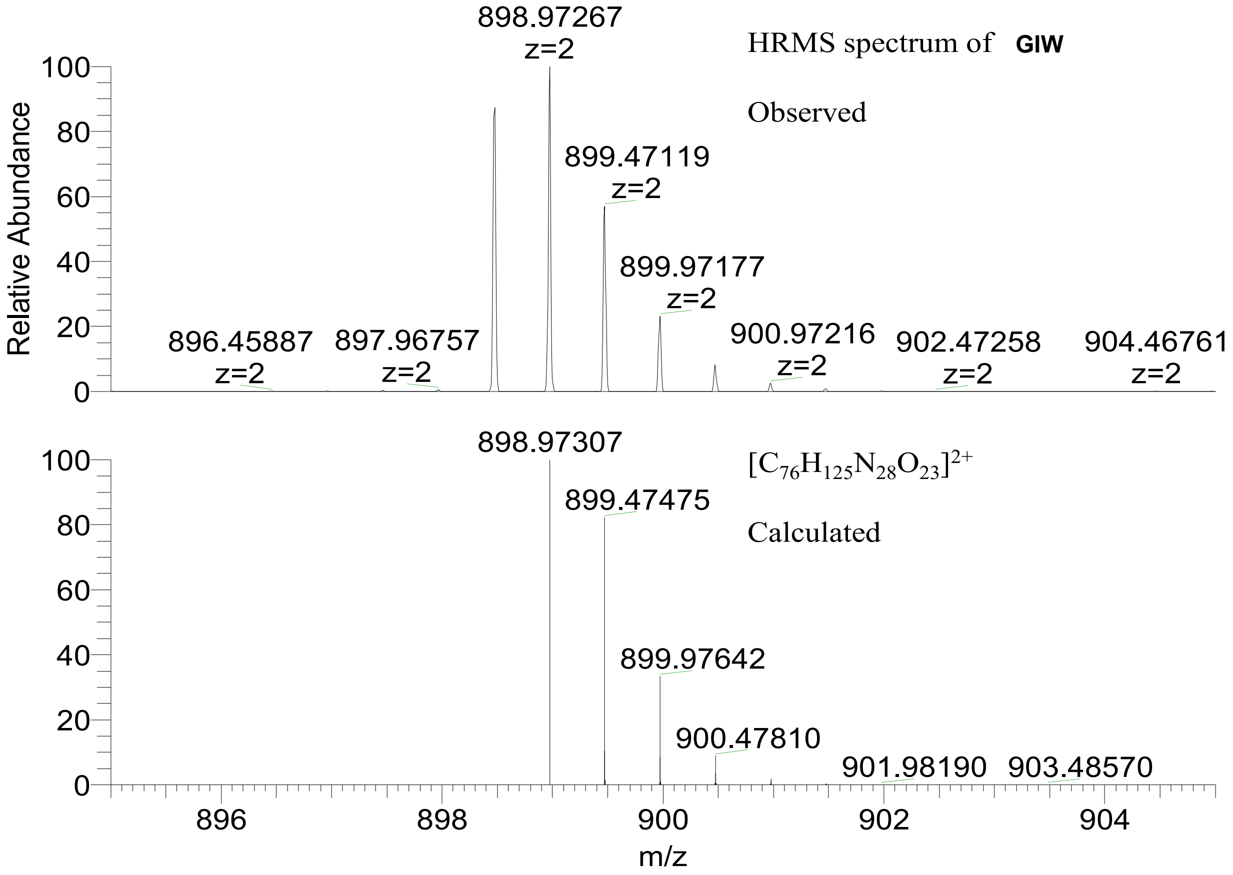

Figure S21. HRMS of the GIW peptide.

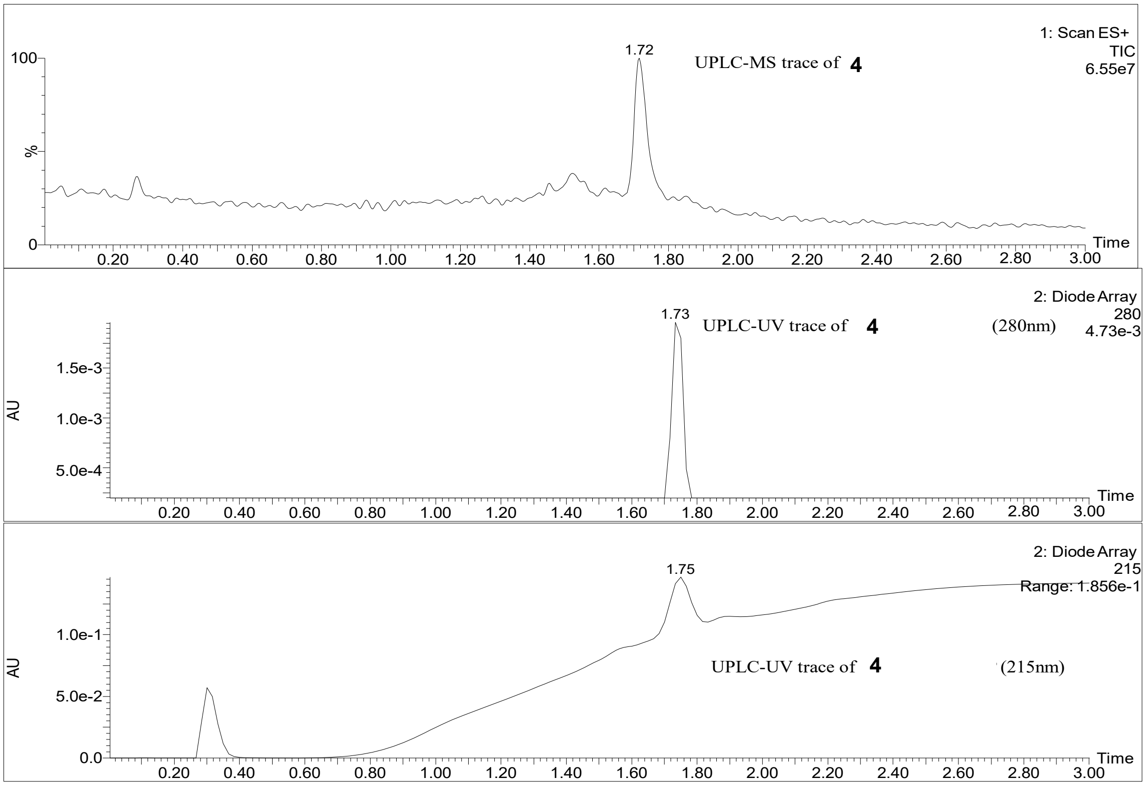

Figure S22. UPLC-MS trace of GIW-bisTCO.

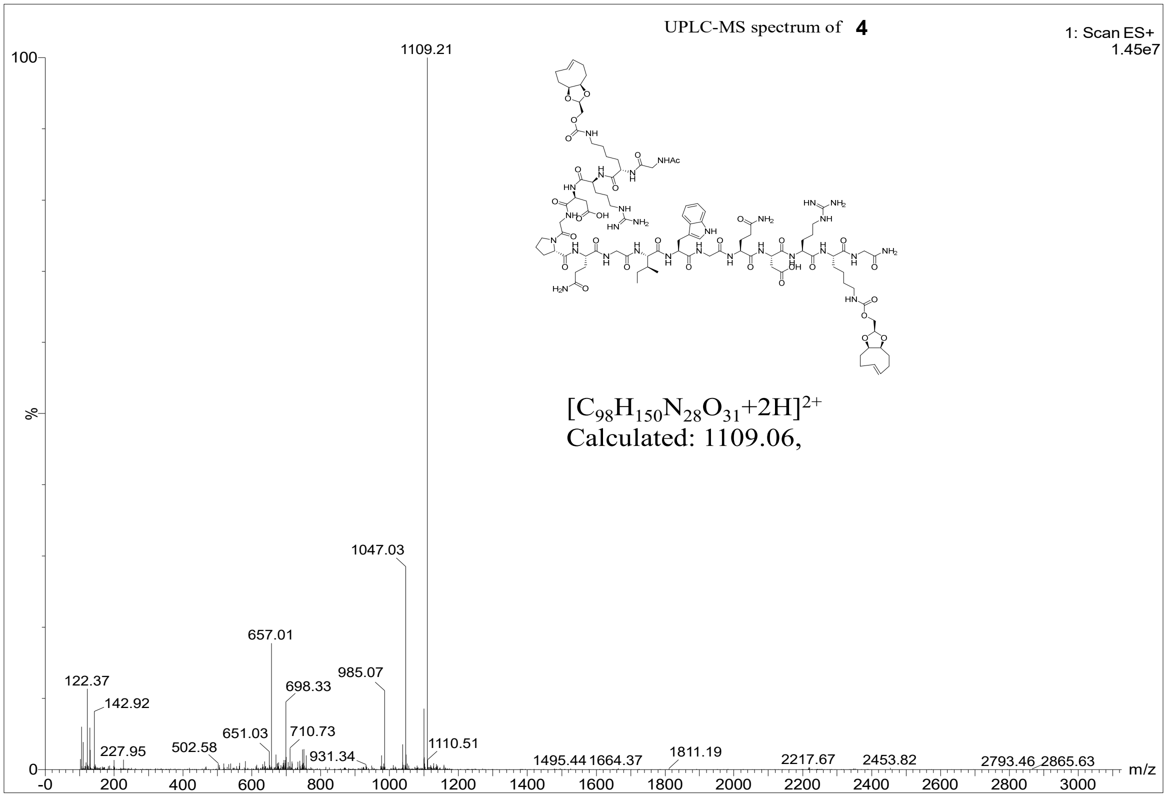

Figure S23. UPLC-MS spectrum of GIW-bisTCO.

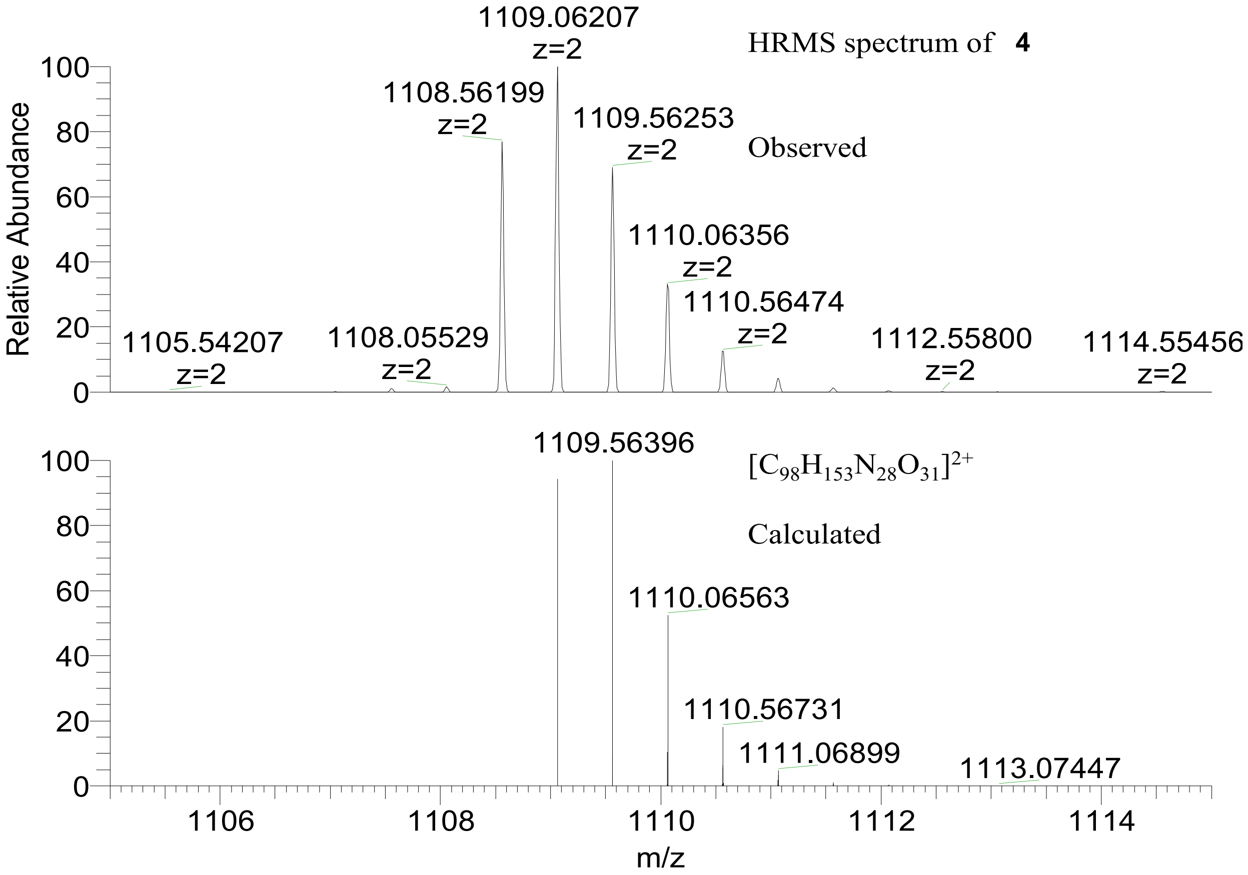

Figure S24. HRMS spectrum of GIW-bisTCO.

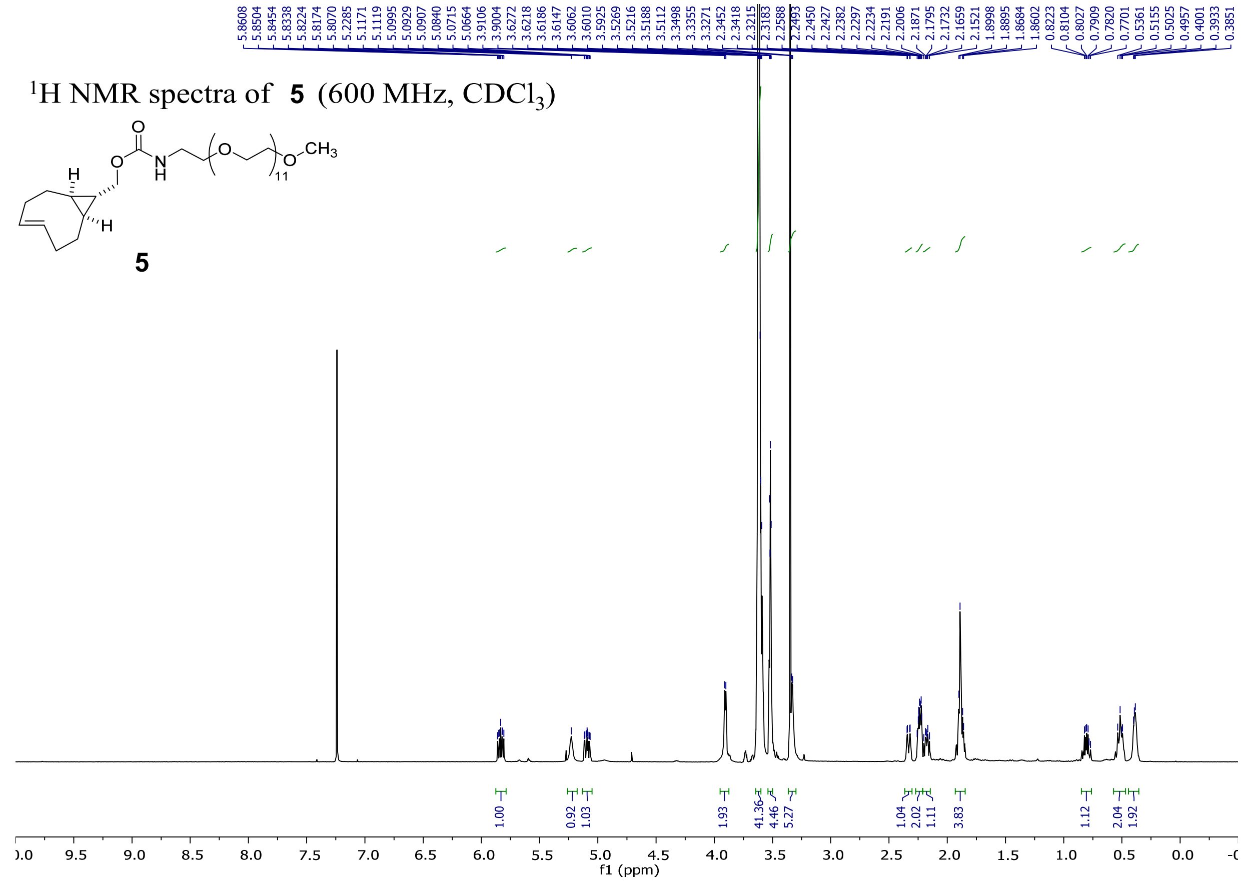

Figure S25. ^{1}H NMR spectrum of PEG-TCO in $CDCl_3$

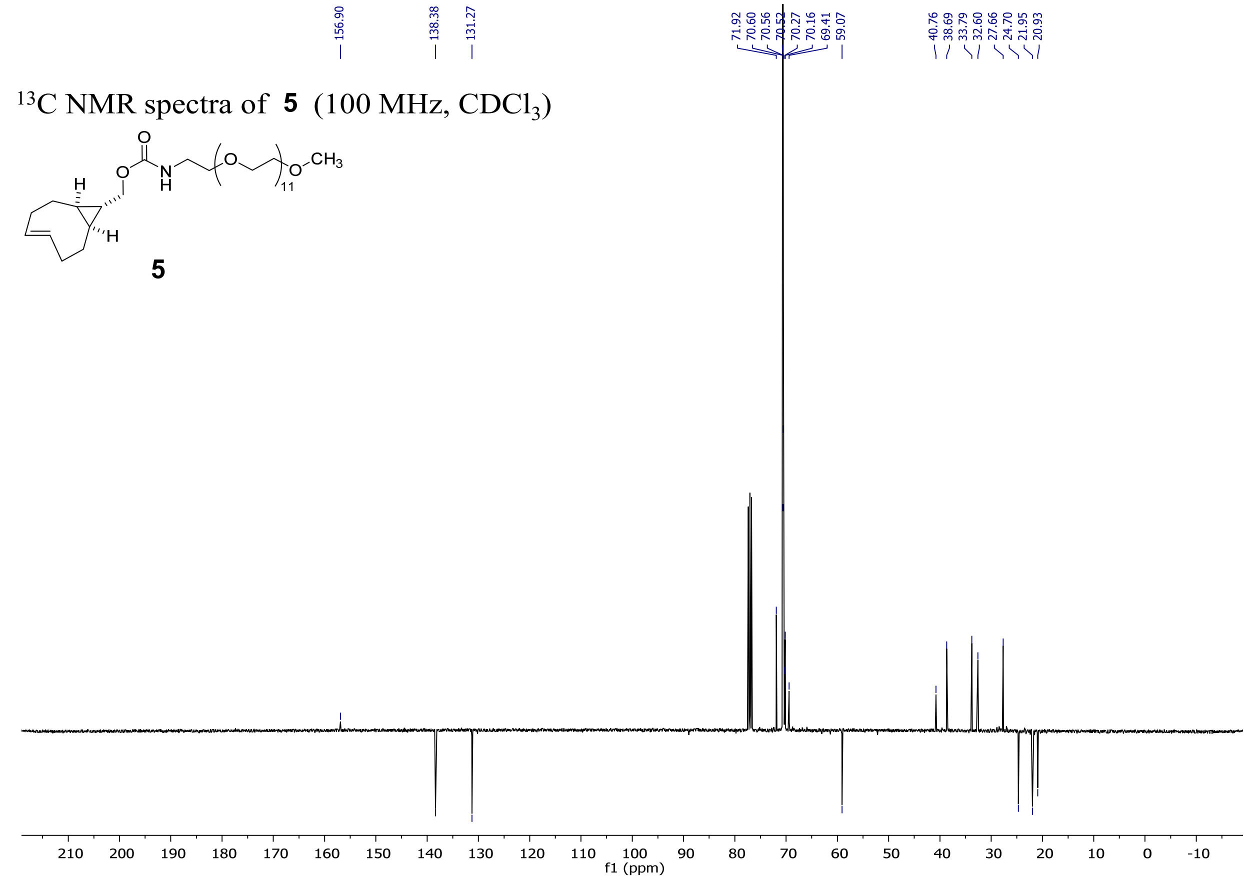

Figure S26. ^{13}C NMR spectrum of PEG-TCO in $CDCl_3$.

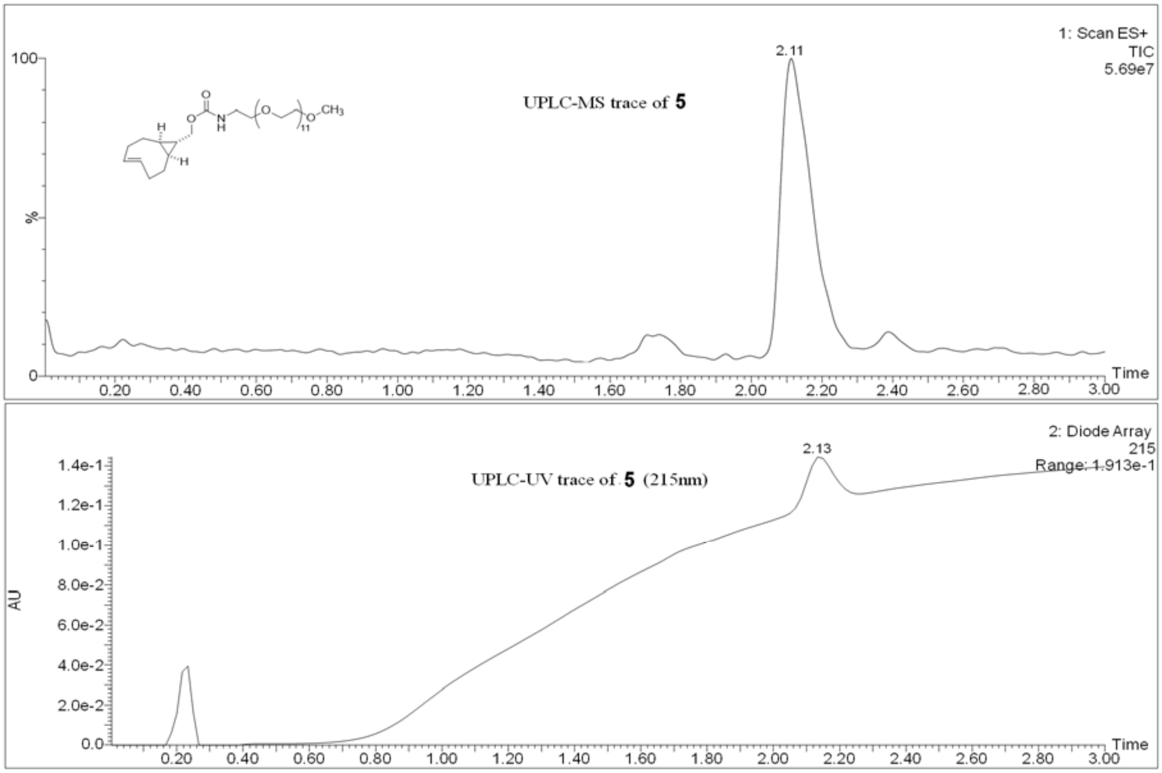

Figure S27. UPLC-MS spectrum of PEG-TCO.

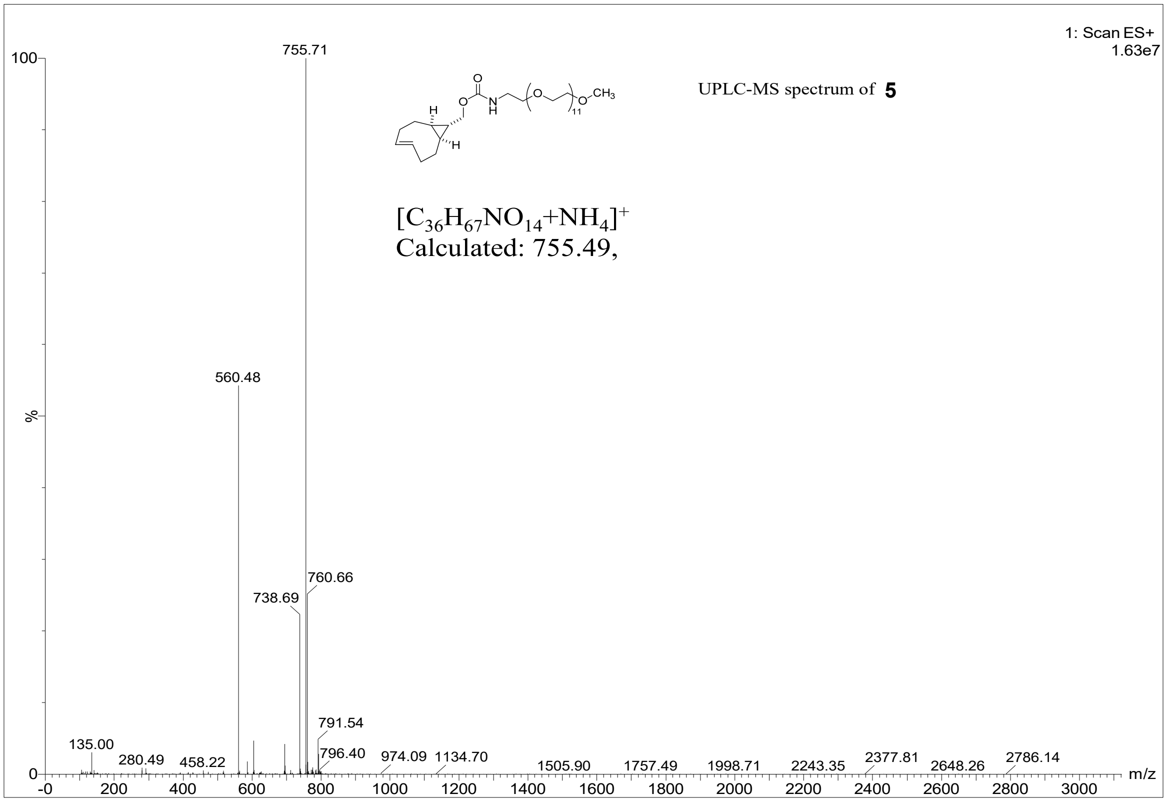

Figure S28. UPLC-MS spectrum of PEG-TCO.

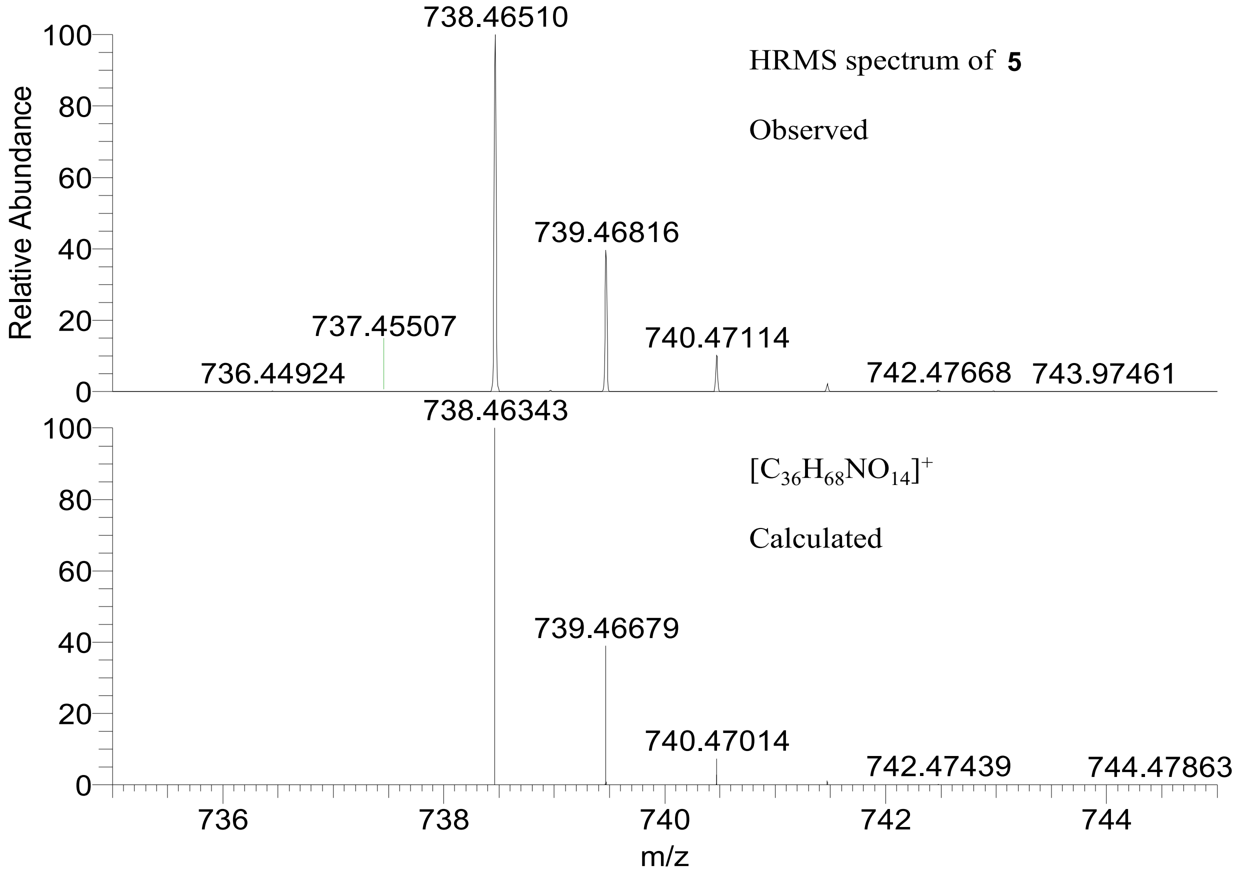

Figure S29. HRMS spectrum of PEG-TCO.

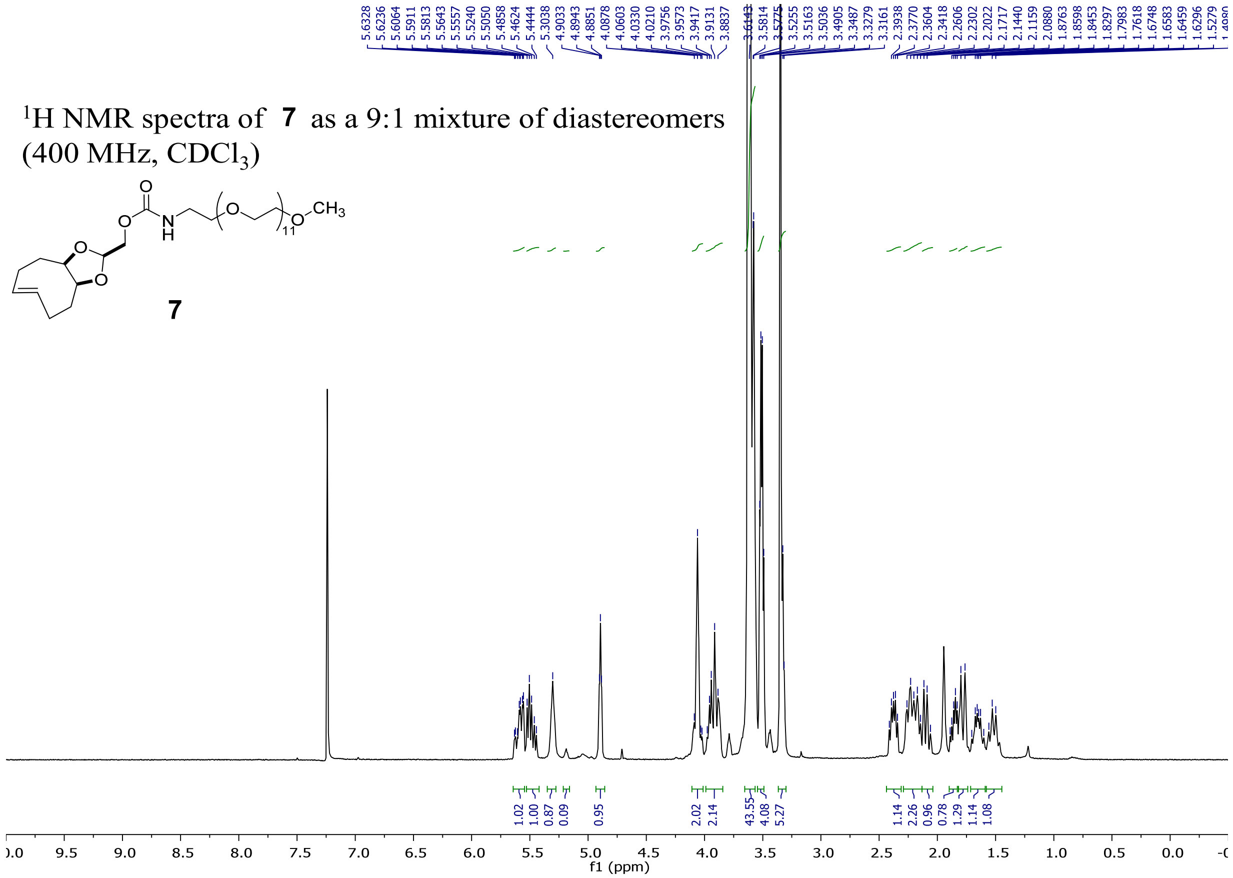

Figure S30. ^{1}H NMR spectrum of PEG-dTCO in $CDCl_3$.

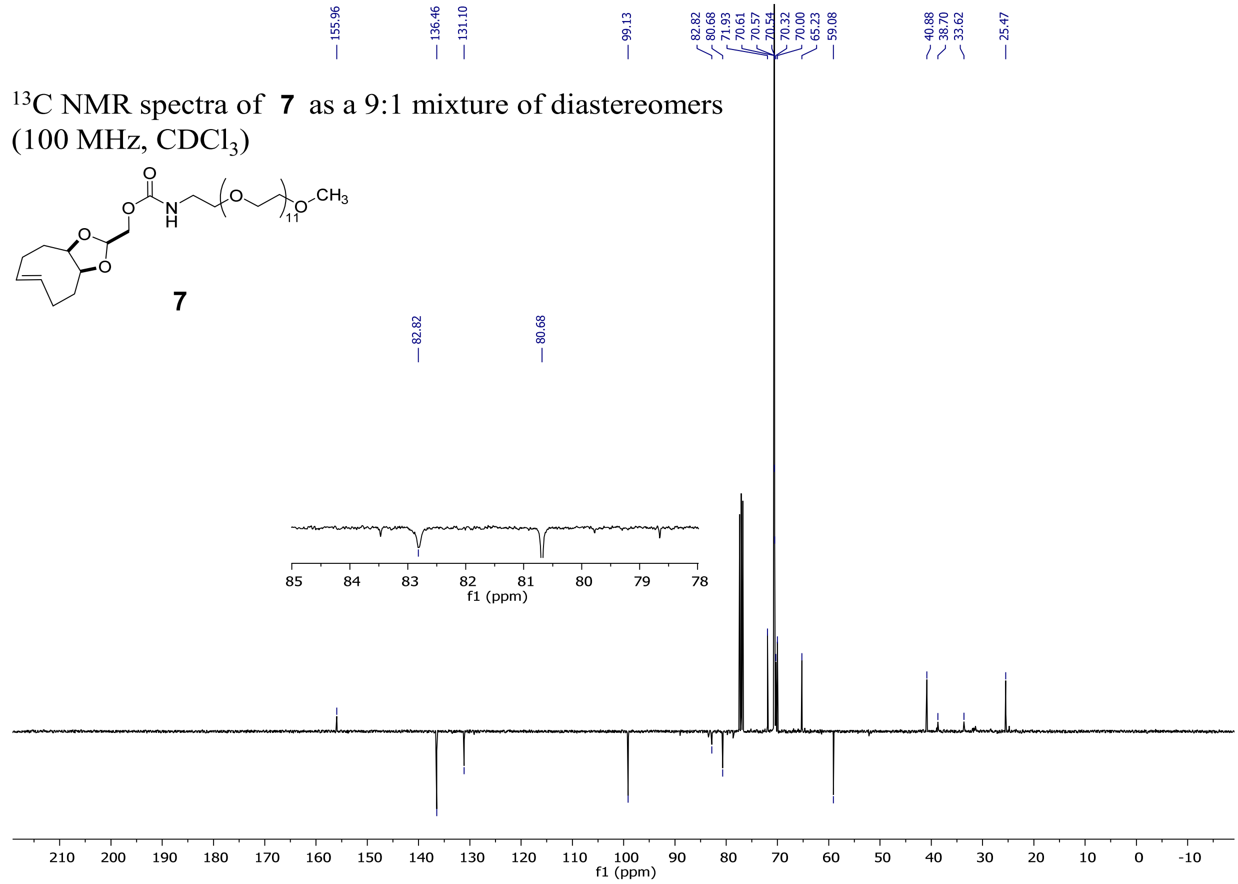

Figure S31. ^{13}C NMR spectrum of PEG-dTCO in $CDCl_3$

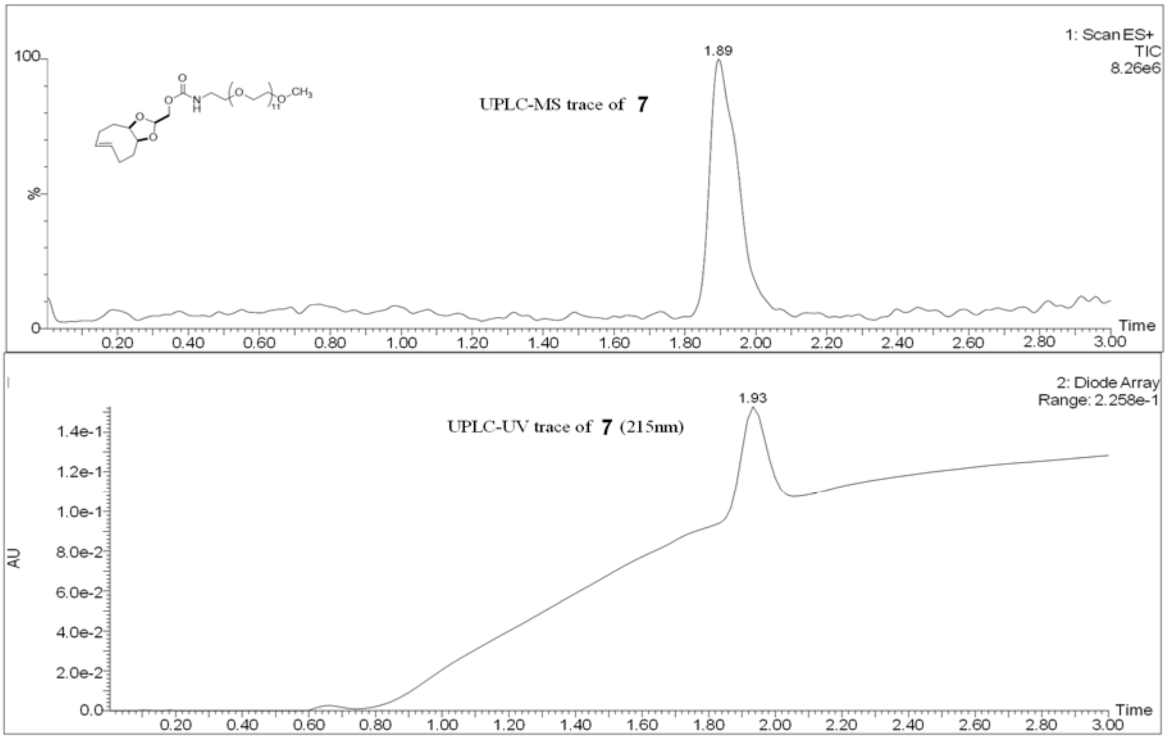

Figure S32. UPLC-MS trace of PEG-dTCO.

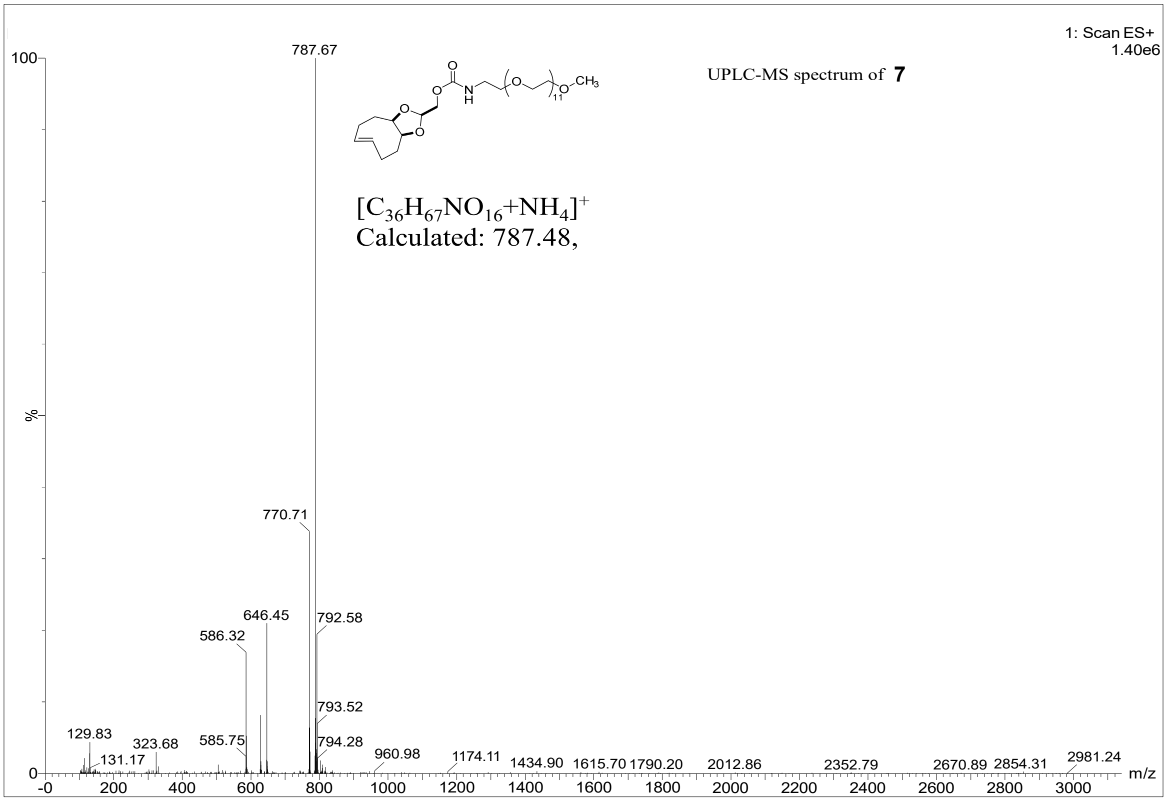

Figure S33. UPLC-MS spectrum of PEG-dTCO.

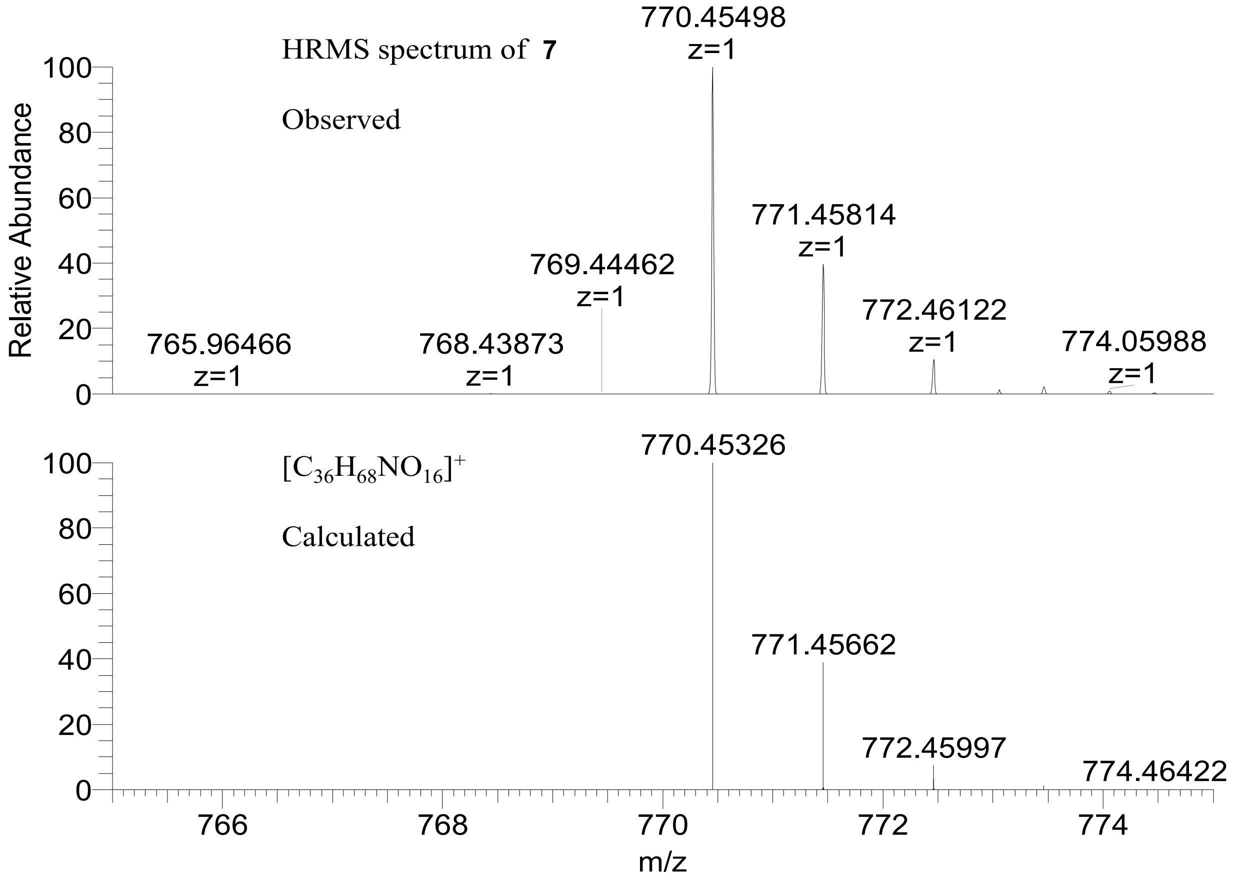

Figure S34. HRMS spectrum of PEG-dTCO.

References:

1 M. T. Taylor, M. L. Blackman, O. Dmitrenko and J. M. Fox, *J. Am. Chem. Soc.*, 2011, **133**, 9646–9649.

2 A. Darko, S. Wallace, O. Dmitrenko, M. M. Machovina, R. A. Mehl, J. W. Chin and J. M. Fox, *Chem. Sci.*, 2014, **5**, 3770–3776.

3 H. Zhang, W. S. Trout, S. Liu, G. A. Andrade, D. A. Hudson, S. L. Scinto, K. T. Dicker, Y. Li, N. Lazouski, J. Rosenthal, C. Thorpe, X. Jia and J. M. Fox, *J. Am. Chem. Soc.*, 2016, **138**, 5978–5983.

4 H. Zhang, K. T. Dicker, X. Xu, X. Jia and J. M. Fox, *ACS Macro Lett.*, 2014, **3**, 727–731.

5 J. J. Young, K. M. Cheng, T. L. Tsou, H. W. Liu and H. J. Wang, *J. Biomater. Sci. Polym. Ed.*, 2004, **15**, 767–780.

6 X. Xu, L. a Gurski, C. Zhang, D. a Harrington, M. C. Farach-Carson and X. Jia, *Biomaterials*, 2012, **33**, 9049–60.
